# A systematic review and meta-analysis of comparative clinical studies on antibiotic treatment of brucellosis

**DOI:** 10.1038/s41598-024-69669-w

**Published:** 2024-08-16

**Authors:** Sachith Maduranga, Braulio Mark Valencia, Xiaoying Li, Samaneh Moallemi, Chaturaka Rodrigo

**Affiliations:** 1 School of Biomedical Sciences, Faculty of Medicine and Health, UNSW Sydney, Sydney, NSW Australia; 2 Kirby Institute, Faculty of Medicine and Health, UNSW Sydney, Sydney, NSW Australia

**Keywords:** Brucellosis, Antibiotics, Systematic review, Treatment, Relapse, Bacterial infection, Clinical microbiology

## Abstract

Brucellosis is a difficult to treat infection that requires antibiotic combinations administered over several weeks for clearance of infection and relapse prevention. This systematic review summarizes current evidence for antibiotic treatment of human brucellosis. PubMed, EMBASE, Scopus, CINAHL, Web of Science, and China Academic Journal databases were searched for prospective studies that had compared different antibiotic regimens for treating human brucellosis in the last 25 years. Thirty-four studies recruiting 4182 participants were eligible. Standard dual therapy with doxycycline + rifampicin had a higher risk of treatment failure compared to triple therapy which added streptomycin (RR: 1.98, 95% CI 1.17–3.35, p = 0.01) or levofloxacin (RR: 2.98, 95% CI 1.67–5.32, p = 0.0002), but a similar or lower risk compared to alternative dual antibiotic combinations (p > 0.05). The same combination had a higher risk of relapses compared to triple therapy which added streptomycin (RR: 22.12, 95% CI 3.48–140.52, p = 0.001), or levofloxacin (RR: 4.61, 95% CI 2.20–9.66, p < 0.0001), but a similar or lower risk compared to other dual antibiotic combinations (p > 0.05). Triple antibiotic therapy is more effective than standard dual therapy with rifampicin and doxycycline. However, the latter is also efficacious and suitable for uncomplicated disease.

## Introduction

Human brucellosis caused by pathogenic^1^ gram-negative coccobacilli of *Brucella spp*. A recent classification also includes *Ochrobactrum* species (pathogenic to immunocompromised humans) within the genus *Brucella*^2^*,* but others have pointed out that they are best placed as a separate genus^3^. This review only focusses on human brucellosis caused by the four pathogenic species *Brucella melitensis, B. suis, B. abortus, and B. canis.* It is one of the most common zoonosis in the world with 2.4 billion people at risk of infection and nearly 0.5–2.1 million new cases reported annually according to different estimates^4,5^. Most of the global burden of brucellosis is shared by countries in the Mediterranean, Central Asia, Middle East, South Asia, North Africa, and Latin America^6^. The infection is typically transmitted to humans via consumption of raw and unpasteurized dairy products, via contact of infected and contaminated animal tissue (an occupational hazard of veterinarians, farmers, abattoir workers) or via inhalation of aerosols^1^. Person to person transmission of brucellosis is rare ^7^. Typical clinical syndrome of human brucellosis is one of prolonged fever (undulant fever) with night sweats, arthralgia, malaise, weight loss, abdominal pain alongside hepatosplenomegaly and lymphadenopathy. Complications include osseo-articular disease, neuro-brucellosis, cardiac involvement (e.g. endocarditis), and genitourinary disease (orchitis, epididymitis and prostatitis)^1^.

Treatment of brucellosis requires combinations of antibiotics administered over an extended period. This converts to more time off work and hence a higher burden of disability adjusted life years lost (DALY) compared to other zoonoses with a global distribution^8^. However, DALY in brucellosis may be underestimated due to limited diagnostic facilities, incomplete epidemiological surveillance, and limited access to treatment in low-middle-income countries. Relapses in brucellosis are observed in 5–10%^9^ of cases though some reports suggest this proportion could be as high as 30%^10^. Relapse is defined as clinical recurrence of disease and elevated titres of *Brucella* specific antibodies after an initial response to treatment. Relapses typically occur within 6 months of initial treatment response but there is no fixed definition for this as some studies have observed patients for longer than 6 months and still reported relapses^11,12^. Relapses are usually due to improper choice of antibiotics, inadequate duration of therapy, poor compliance, or a combination of these factors. It is unlikely to be due to antibiotic resistance, which should manifest as primary treatment failure.

Given the risk of complications and poor quality of life associated with ongoing infections, it is important to avoid treatment failure and relapses with efficacious combinations of antibiotics based on current evidence. The aim of this systematic review is to summarise current evidence for antibiotic treatment of human brucellosis as shown in prospective controlled human clinical studies. The last comprehensive systematic reviews on this topic were published more than a decade ago in 2012/2013^13,14^ and several clinical trials and comparative studies had been published afterwards. Another systematic review was published in 2023^15^ while ours was in progress, but it only compared triple antibiotic combinations against dual combinations for brucellosis. Our review is more comprehensive as it considers comparisons of all antibiotic combinations regardless of whether they are triple, dual combinations or monotherapy. This review also considers comparisons of different durations and dosages of the same antibiotic combination.

## Methods

### Study eligibility: participants, intervention, comparator and outcomes

Comparative prospective clinical studies that assessed the efficacy of antibiotics in treatment of human brucellosis were eligible. The participants were adults and children with clinical features suggestive of brucellosis with at least one laboratory test result supportive of infection (bacterial culture, polymerase chain reaction, serological evidence, or a combination of these). Randomized, non-randomized clinical trials as well as prospective observational studies that simultaneously followed up two groups of patients treated differently were included if separate groups were treated with different antibiotics or with the same antibiotic/s but in different durations or dosages. Studies that evaluated the efficacy of treatments other than antibiotics (e.g. antioxidants, hydroxychloroquine) were excluded if the antibiotic treatment were similar across groups. Retrospective studies (including case control studies) were also excluded to preserve the quality of evidence and to reduce the risk of bias (e.g. recall and selection bias). Outcomes assessed were the numbers of initial cure and relapses during follow-up.

### Search strategy

We searched PUBMED, Scopus, Web of Science, CINAHL, EMBASE and the CNKI (China Academic Journals) with search term “(brucellosis OR brucella) AND (treat* OR intervention) AND (prospective OR random* OR control* OR trial OR cohort)” in title, abstract or keywords (Supplementary Table 1). Articles were limited to those published within 25 years (after 1997) to maintain recency of evidence. There were no limits based on language of publication. The last date of search was 6th June 2023. Bibliographies of eligible articles were also searched. Abstract screening, and full-text searches were done by SM (Sachith), BMV and CR independently after importing the search results from each database to Covidence (https://www.covidence.org/). Additionally full-text articles in Spanish, Persian and Mandarin were read by BMV, SM (Samaneh), and XL respectively. Retrospective studies, animal and in vitro studies, narrative and systematic reviews, opinion papers and editorials were excluded. Any disagreements were resolved by consensus.

### Data extraction and analysis

From the eligible studies following data items were extracted: study location, inclusion criteria, diagnostic criteria, dosage of antibiotics in each treatment arm, definitions for treatment failure/relapse, number of events in each treatment arm, and severe adverse events. Severe adverse events were those that led to hospital admission, treatment stoppage or death. Only published data were used. The effect size of each treatment comparison was expressed as risk ratios with 95% confidence intervals. If two or more studies were similar in terms of participants, interventions, comparators and outcomes, meta-analyses were performed using a fixed effect model. Heterogeneity was assessed with the I^2^ statistic (> 70%)^16^, and in event of high heterogeneity or when the results were likely to be influenced by sample size differences, the analysis was repeated with a random effects model. All meta-analyses were initially done with an intention to treat analysis and a sensitivity analysis was performed using a per protocol approach. Subgroup analyses were not planned. All meta-analyses were visually displayed with Forest plots. Data extraction was done by CR and SM (Sachith) independently (rechecked by BMV), and the data analysis was done by CR (rechecked by others) using the Revman software (Version 5.4)^17^. The protocol for this review is registered in PROSPERO (https://www.crd.york.ac.uk/prospero, Reference: CRD42023453290).

### Risk of bias and certainty of evidence

The risk of bias in each eligible study was assessed using the Cochrane risk of bias assessment tool for intervention studies^17^. The certainty of evidence was assessed according to GRADE recommendations^18^.

## Results

**Figure 1:**
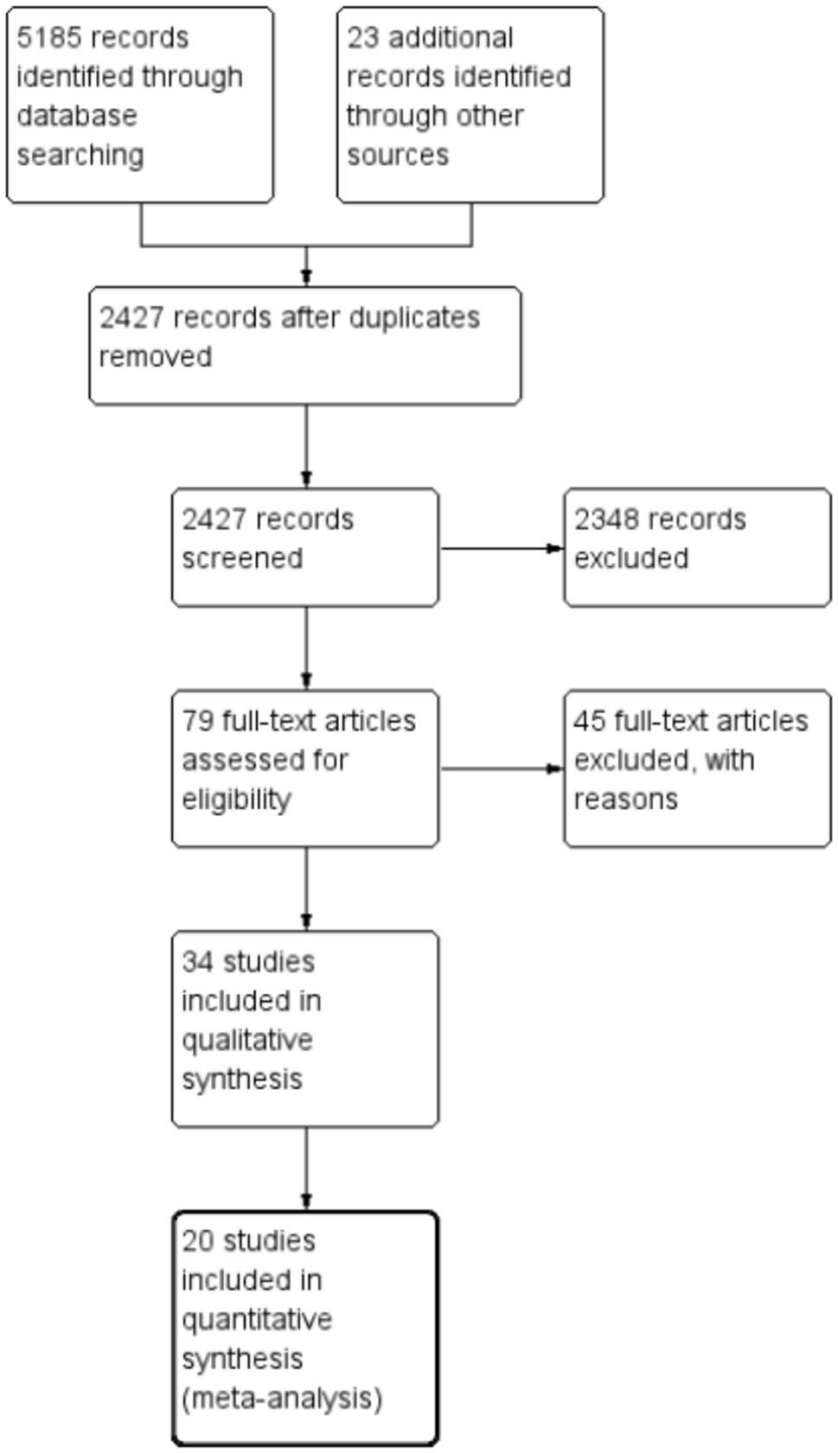
PRISMA flow chart of study selection.

Thirty-four studies^19–52^ published between 1999 and 2022 recruiting 4182 patients met the inclusion criteria. A further 44 studies were excluded with reasons after reviewing full-text publications (Fig. 1). The number of patients in eligible studies ranged from 34 to 339. Most of the studies were from Iran (n = 14)^20,21,24,28–30,32,33,35–37,39,41,42^, China (n = 10)^43–52^, and Turkey^19,22,23,26,30,40^ (n = 6) while Saudi Arabia^25^, North Macedonia^34^, Egypt^27^, Spain^38^ had one study each. All studies recruited adults though the lower age limit for “adults” varied between 14 and 21 years. Six studies^31,34–37,42^ recruited children also and the upper age limit for a child varied from 8 to 12 years across these studies. Diagnostic tests for brucellosis varied across studies from using blood culture alone, measuring *Brucella* specific antibodies and their titres (e.g. serum agglutination test, 2-mercaptoethanol agglutination test, ELISA for serum IgG) in patients with clinical symptoms or a combination of these methods (Supplementary Table 2). Four studies^25,26,29,32^ compared 3 antibiotic regimens, one study^23^ compared five antibiotic regimens and all others compared two antibiotic regimens within their trial arms. Each antibiotic regimen within a trial arm had a minimum of one antibiotic and a maximum of three antibiotics and were administered for a minimum of 30 days and a maximum of 84 days (Supplementary Table 2). The most used antibiotic combination was doxycycline with rifampicin. In addition, streptomycin, cotrimoxazole, sulfamethoxazole, ofloxacin, ciprofloxacin, levofloxacin, streptomycin, gentamycin, amikacin and tetracycline were also used as different combinations in comparisons. Most studies reported on two outcomes; treatment failure (recurrence of symptoms or rising antibody titres during treatment after a short period of clinical response) and relapse (recurrence of signs/ symptoms with either serological or culture evidence of infection during follow up after successful treatment). The follow up period after completion of therapy varied from 6 to 24 months between the studies. Characteristics of all included studies are further detailed in Supplementary Table 2. Studies that were excluded after full-text review are listed with reasons for exclusion in Supplementary Table 3.

The types of antibiotics compared, their doses and duration of treatment were heterogenous across studies. Furthermore, in trials that included both children and adults, the children had doses calculated according to bodyweight while adults mostly received fixed dose combinations. Thus, for purposes of meta-analysis we only considered similar combinations of antibiotics without an exact match for dose and duration of treatment as matching all parameters was impossible. Given that doses of antibiotics were mostly similar except for body weight related adjustments (Supplementary Table 2), this approach is justified. Also, if differences in duration of treatment had a significant effect on meta-analyses it would have increased the heterogeneity (I^2^ > 70%) in results, which was not observed in any of the comparisons.

### Risk of bias

**Figure 2:**
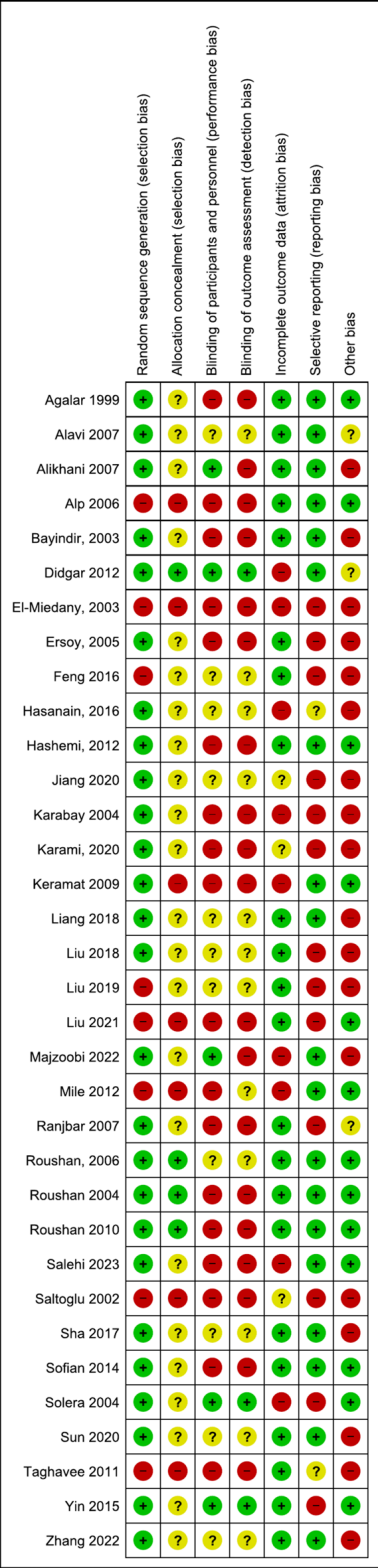
Risk of bias summary for included studies.

Apart from eight studies^22,25,34,39,40,48,50,51^ all others reported random allocation of participants to each study arm. However, only 4 studies^24,28,36,37^ described allocation concealment within methods (Fig. 2). Three studies^24,38,43^ were double blinded while only the patients were blinded in another two studies^21,33^. All other studies had a high risk of detection or performance bias or both. Nine studies had greater than 10% of randomized participants lost to follow up (high attrition bias)^24,25,27,30,32–34,38,41^. The attrition was mostly during follow up for relapses after treatment completion. Thirteen studies^25,26,30,31,35,38,40,43,46,48,50–52^ had potential reporting bias because they either did not account for all patients randomized, did not report the number of initial treatment failures or or did not report on relapses. A detailed breakdown of potential sources of bias for each study is given in Supplementary Table 4.

### Risk of treatment failure

**Figure 3:**
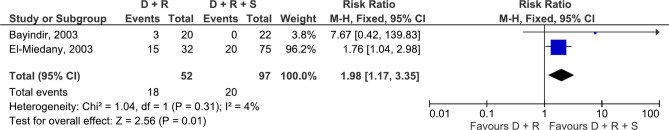
Forest plot of meta-analysis. Doxycycline (D) + Rifampicin (R) vs. Doxycycline (D) + Rifampicin (R) + Streptomycin (S), outcome: treatment failure.

**Figure 4:**
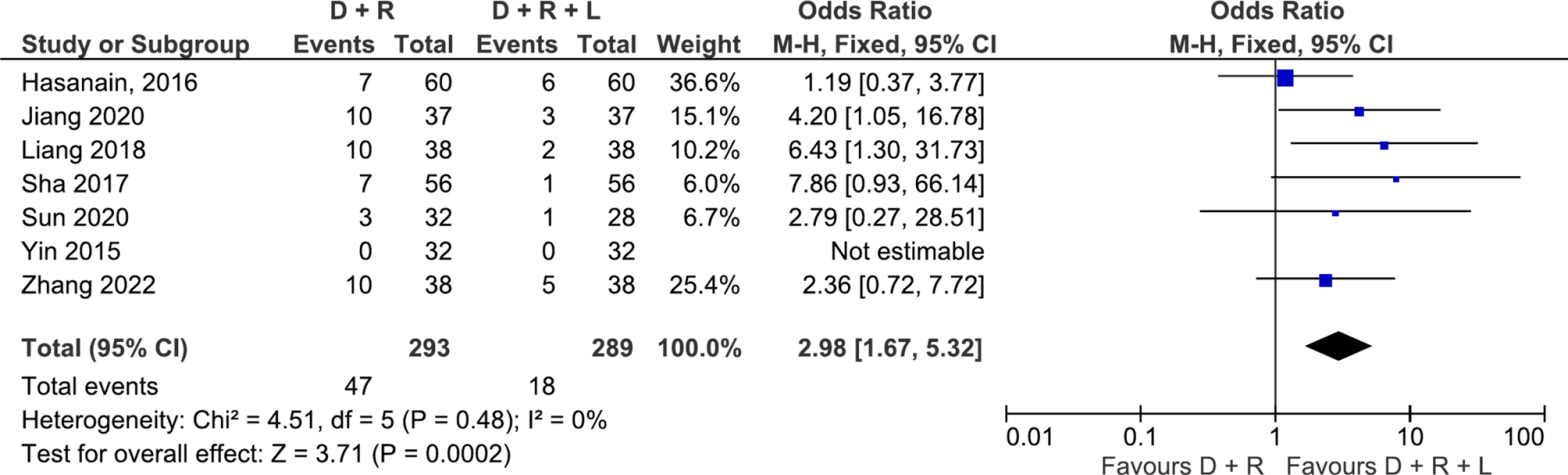
Forest plot of meta-analysis. Doxycycline (D) + Rifampicin (R) vs. Doxycycline (D) + Rifampicin (R) + Levofloxacin (L), outcome: treatment failure.

**Figure 5:**
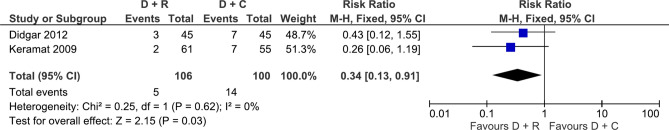
Forest plot of meta-analysis. Doxycycline (D) + Rifampicin (R) vs. Doxycycline (D) + Ciprofloxacin (C), outcome: treatment failure.

Nine antibiotic comparisons were assessed in 2 or more studies for the outcome of treatment failure making them eligible for meta-analyses. Regarding risk of treatment failure, doxycycline, rifampicin, streptomycin triple combination outperformed doxycycline, rifampicin dual combination (RR: 1.98, 95% CI 1.17–3.35, 2 studies, 149 participants, p = 0.01, low certainty, Fig. 3)^23,25,28,36,37^. However, the two eligible studies for this comparison were different in sample sizes and when re-analysed with a random effects model which is less influenced by sample size, the result became statistically insignificant (p = 0.05). In addition, doxycycline, rifampicin and levofloxacin (oral or intravenous) triple combination also outperformed the doxycycline and rifampicin dual combination (RR: 2.98, 95% CI 1.67–5.32, 7 studies, 582 participants, p = 0.0002, moderate certainty, Fig. 4)^27,43–47,49^. When comparing dual combinations, doxycycline plus rifampicin combination was significantly better than doxycycline plus ciprofloxacin combination (RR: 0.34, 95% CI 0.13–0.91, 2 studies, 206 participants, p = 0.03, moderate certainty, Fig. 5)^24,32^, and as efficacious as three other antibiotic combinations (ciprofloxacin + rifampicin^19,32,39^, ofloxacin + rifampicin^23,26,29^, doxycycline + streptomycin^23,26,29^) (Supplementary Figs. 3, 5, 6, 9, 11, 12). Two studies compared doxycycline plus rifampicin dual combination against doxycycline only and found the dual combination to be superior (RR: 0.19, 95% CI 0.06–0.6, 2 studies, 171 participants, p = 0.005, moderate certainty, Supplementary Fig. 14)^48,52^.

There were many other antibiotic comparisons that had only been assessed in a single study and hence could not be combined in a meta-analysis. Their results are summarized in Table 1.

**Table 1:** Risk of treatment failure with different antibiotic regimens (for comparisons that could not be included in a meta-analysis, intention to treat analysis).

Study	Treatment 1*	Events (treatment failure)	Denominator	Treatment 2*	Events	Denominator	RR (95% CI)	P value
Alavi et al. 2007^20^	D + R	5	52	D + Co	1	53	5.09 (0.61–42.15)	0.113
Alikhani et al. 2007^21^	D + R + S	0	37	O + S + R	0	38	N/A	N / A
Alp et al. 2006^22^	D + S	0	15	C + R	0	16	N/A	N / A
Bayindir et al.^#^ 2003^23^	D + R	3	20	S + T	2	20	1.5 (0.28–8.04)	1.000
	D + S + R	0	22	S + T	2	20	N/A	0.221
	D + S + R	0	22	D + S	4	21	N/A	0.049**
	D + S + R	0	22	O + R	5	19	N/A	0.016**
	O + R	5	19	S + T	2	20	2.63 (0.56–11.97)	0.235
	O + R	5	19	D + S	4	21	1.38 (0.43–4.4)	0.711
El-Miedany et al. 2003^25^	D + R	0	32	R + Co	0	32	N/A	N/A
	D + S + R	0	75	R + Co	0	32	N/A	N/A
Hasanain et al. 2016^27^	D + R	7	60	D + R + L	6	60	1.17 (0.42–3.27)	1.000
Keramat et al. 2009^32^	C + R	3	62	D + C	7	55	0.38 (0.1–1.4)	0.187
Liu et al. 2019^51^	D + R	7	60	D + R + Sm	0	64	N/A	0.005
Liu et al. 2021^50^	D + R	9	36	D + L	2	36	4.5 (1.04–19.39)	0.046
Majzoobi et al. 2022^33^	D + S + H (Short duration)	8	46	D + S + H (long duration)	4	46	2 (0.65–6.18)	0.216
Mile et al. 2012^34^	D + R + G	5	113	D + R	5	125	1.11 (0.33–3.72)	1.000
Roushan et al. 2004^37^	D + Co	5	140	R + Co	13	140	0.38 (1.14–1.05)	0.051
Salehi et al. 2023^41^	D + R (low dose)	11	65	D + R (high dose)	3	65	3.67 (1.07–12. 54)	0.024**
Sofian et al. 2014^42^	D + R + S (short duration)	0	79	D + R + S (long duration)	0	79	N/A	N/A

*Doses and duration of treatment are detailed in Table 1 (D—Doxycycline, R—Rifampicin, S—Streptomycin, Co—Cotrimoxazole, G—Gentamycin, A—Amikacin, L—Levofloxacin, O—Ofloxacin, C—Ciprofloxacin, Sm—Sulfamethoxazole, H—Hydroxychloroquine which is not an antibiotic), **p < 0.05 (Fisher exact probability test used when appropriate), ^#^This study had five different treat regimens and hence 20 possible pair-wise comparisons—Only comparisons against commonly used D + R, O + R and D + R + S regimens (not included in meta-analyses) are shown in this table.

### Risk of relapse

**Figure 6:**
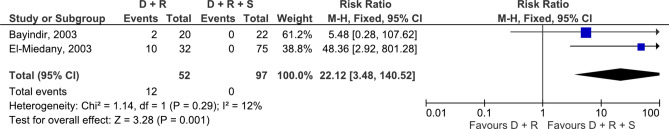
Forest plot of meta-analysis. Doxycycline (D) + Rifampicin (R) vs. Doxycycline (D) + Rifampicin (R) + Streptomycin (S), outcome: relapse.

**Figure 7:**
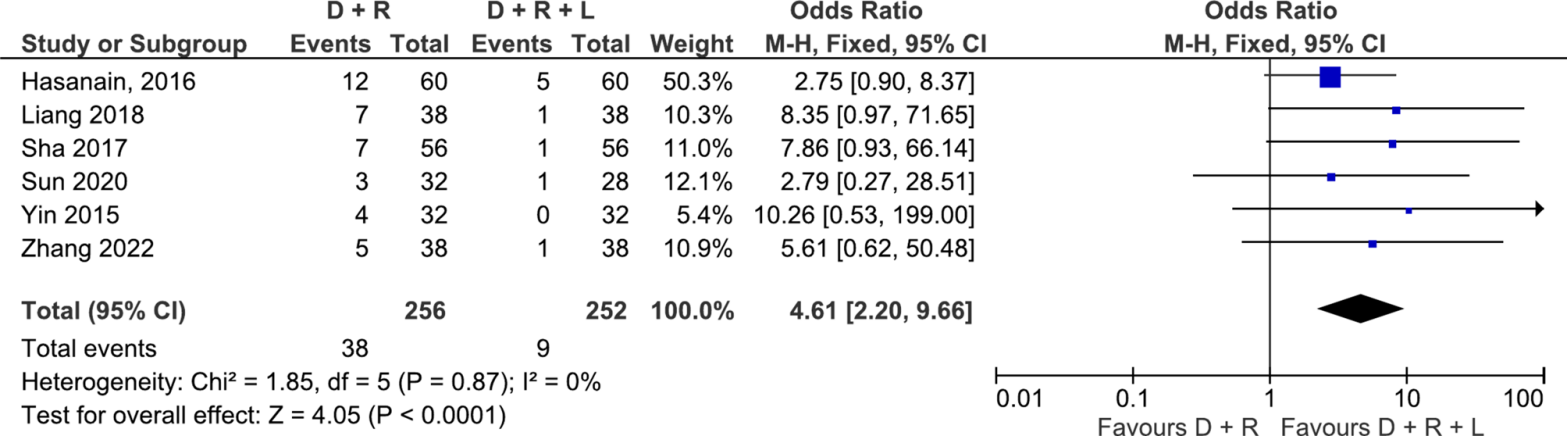
Forest plot of meta-analysis. Doxycycline (D) + Rifampicin (R) vs. Doxycycline (D) + Rifampicin (R) + Levofloxacin (L), outcome: relapse.

**Figure 8:**
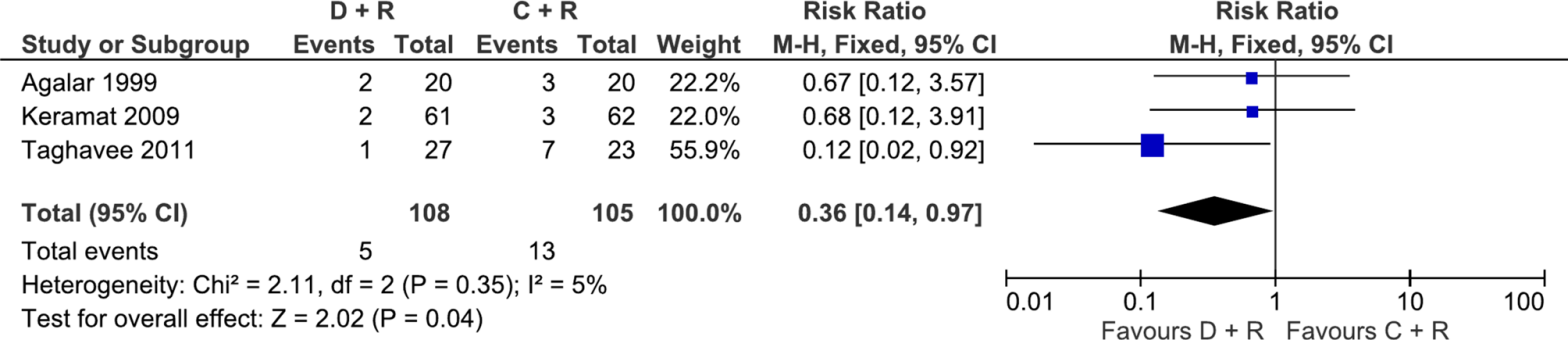
Forest plot of meta-analysis. Doxycycline (D) + Rifampicin (R) vs. Ciprofloxacin (C) + Rifampicin (R), outcome: relapse.

**Figure 9:**
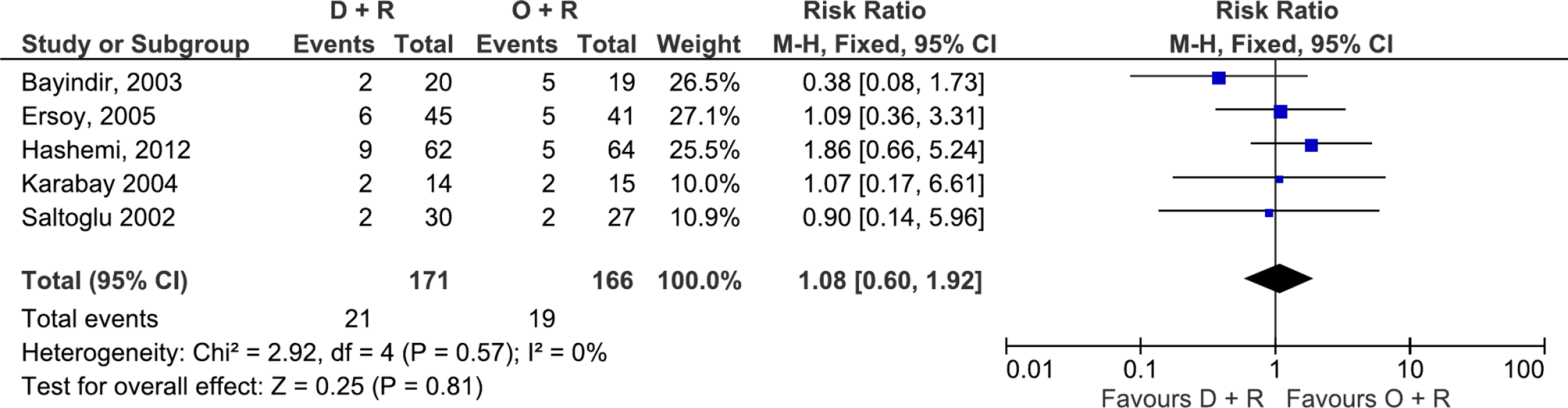
Forest plot of meta-analysis. Doxycycline (D) + Rifampicin (R) vs. Ofloxacin (O) + Rifampicin (R), outcome: relapse.

Seven different antibiotic comparisons had been compared for their efficacy in relapse prevention in two or more similar studies enabling several meta-analyses. For this outcome doxycycline, rifampicin, streptomycin triple combination outperformed doxycycline plus rifampicin dual combination (RR: 22.12, 95% CI 3.48–140.52, 2 studies, 149 participants, p = 0.001, moderate certainty, Fig. 6)^23,25^ when analysed with either fixed or random effects models. The alternative triple therapy of doxycycline, rifampicin plus levofloxacin also outperformed doxycycline plus rifampicin dual combination (RR: 4.61, 95% CI 2.20–9.66, 6 studies, 508 participants, p < 0.0001, moderate certainty, Fig. 7)^27,43–45,47,49^. As for comparisons between dual therapies, the most commonly used doxycycline plus rifampicin combination was superior to the ciprofloxacin plus rifampicin combination (RR: 0.36, 95% CI 0.14–0.97, 3 studies, 206 participants, p = 0.04, moderate certainty, Fig. 8)^19,32,39^, but inferior to the doxycycline plus streptomycin combination (RR: 2.37, 95% CI 1.01–5.55, 2 studies, 264 participants, p = 0.05, moderate certainty)^23,26,29^ and had the same efficacy as ofloxacin plus rifampicin combination (5 studies, 337 participants, p > 0.05, high certainty, Fig. 9) ^23,26,29,30,40^. The meta-analyses results for all other comparisons for relapse prevention are shown in Supplementary Figs. 2, 4, 7, 8, 10 and 13. The results for antibiotic regimens that were only assessed in a single study are summarized in Table 2.

**Table 2:** Risk of relapse with different antibiotic regimens (for comparisons that could not be included in a meta-analysis, intention to treat analysis).

Study	Treatment 1*	Events	Denominator	Treatment 2*	Events	Denominator	RR (95% CI)	P value
Alavi et al. 2007^20^	D + R	6	52	D + Co	3	53	2.04 (0.54–7.72)	0.319
Alikhani et al. 2007^21^	D + R + S	0	37	O + S + R	0	38	N/A	N/A
Alp et al. 2006^22^	D + S	0	15	C + R	0	16	N/A	N/A
Bayindir et al.^#^ 2003^23^	D + R	2	20	S + T	0	20	N/A	0.487
	D + S + R	0	22	S + T	0	20	N/A	N/A
	D + S + R	0	22	D + S	0	21	N/A	N/A
	D + S + R	0	22	O + R	5	19	N/A	0.016**
	O + R	5	19	S + T	0	20	N/A	0.02**
	O + R	5	19	D + S	0	21	N/A	0.018**
El-Miedany et al. 2003^25^	D + R	10	32	R + Co	11	32	0.91 (0.45–1.83)	1.000
	D + S + R	0	75	R + Co	11	32	N/A	< 0.001**
Hasanain et al. 2016^27^	D + R	12	60	D + R + L	5	60	2.4 (0.9–6.39)	0.067
Karami et al. 2020^31^	D + R + G (short duration)	49	175	D + R + G (long duration)	25	164	1.83 (1.19–2.83)	0.004**
Keramat et al. 2009^32^	C + R	3	62	D + C	7	55	0.38 (0.1–1.39)	0.187
Majzoobi et al. 2022^33^	D + S + H (short duration)	3	46	D + S + H (long duration)	4	46	0.75 (0.18–3.17)	1.000
Mile et al. 2012^34^	D + R + G	4	113	D + R	13	125	0.34 (0.11–1.01)	0.04**
Ranjbar et al. 2007^35^	D + R	9	114	D + R + A	6	114	1.5 (0.55–4.08)	0.424
Roushan et al. 2004^37^	D + Co	12	140	R + Co	14	140	0.86 (0.41–1.79)	0.68
Salehi et al. 2023^41^	D + R (low dose)	9	65	D + R (high dose)	3	65	3 (0.85–10.58)	0.07
Sofian et al. 2014^42^	D + R + S (short duration)	10	79	D + R + S (long duration)	7	79	1.43 (0.57–3.56)	0.442
Solera et al. 2004^38^	D + G (short duration)	15	84	D + G (long duration)	9	83	1.65 (0.76–3.55)	0.196

*Doses and duration of treatment are detailed in Table 1 (D—Doxycycline, R—Rifampicin, S—Streptomycin, Co—Cotrimoxazole, G—Gentamycin, A—Amikacin, L—Levofloxacin, O—Ofloxacin, C—Ciprofloxacin, H—Hydroxychloroquine which is not an antibiotic), **p < 0.05 (Fisher exact probability test used when appropriate), ^#^This study had five different treat regimens and hence 20 possible pair-wise comparisons—Only comparisons against commonly used D + R, O + R and D + R + S regimens (not included in meta-analyses) are shown in this table.

### Serious adverse events

Serious adverse events were relatively rare and were reported in only six studies (Supplementary Table 5)^20,22,26,34,37,42^. Three studies did not report on adverse events^19,25,39^. All remaining studies reported that there were no serious adverse events.

### Sensitivity analysis

When meta-analyses were repeated with a per protocol analysis for both treatment failures and relapses, the results were the same (p < 0.05) for all statistically significant results as those observed with an intention to treat analysis, except for doxycycline + rifampicin vs. ciprofloxacin + rifampicin comparison for relapse prevention where both regimens were equally effective (p > 0.05, 3 studies, 134 participants) instead of the former being better. Also, using anti-*Brucella* IgG ELISA for diagnosis may generate false positives due to cross reactivity with other bacterial antigens (e.g. *E. coli, Yersinia pestis, Vibrio cholerae*). We excluded Hashemi et al.^29^, who had used this test for diagnosis from meta-analyses in a sensitivity analysis and the results did not change for all except one. That is, the doxycycline + streptomycin combination was no longer superior to the doxycycline + rifampicin combination for relapse prevention. Three studies^22,23,48^ had exclusively included patients with osseo-articular complications while one study^25^ had a majority of patients with such complications. Given that patients with complications are treated differently, we performed a sensitivity analysis by excluding these studies from relevant meta-analyses if they had been combined with studies which recruited uncomplicated patients. Except when a meta-analysis was no longer possible, the interpretations changed in only one instance. That is the doxycycline + streptomycin combination was no longer superior to the doxycycline + rifampicin combination for relapse prevention.

## Discussion

This systematic review of controlled clinical trials on antibiotic treatment for brucellosis considered 4182 patients enrolled across 34 studies published after 1997, and concluded that triple therapy with doxycycline and rifampicin plus streptomycin or levofloxacin was superior to the more commonly used dual combination of doxycycline and rifampicin for initial cure (low to moderate certainty evidence) and relapse prevention (moderate certainty evidence). In all other meta-analyses doxycycline and rifampicin combination was superior to or had similar efficacy to all other antibiotic combinations tested (except against doxycycline plus streptomycin combination for relapse prevention).

Most trials used doxycycline plus rifampicin combination as the “standard” to compare against a different antibiotic regimen and this is justified given that only triple combinations outperformed it in meta-analyses. However, streptomycin is typically administered intramuscularly (painful), or less commonly intravenously (requires hospitalization or frequent hospital visits as an outpatient) which makes it a less favourable option than the fully orally administrable doxycycline + rifampicin combination. If triple therapy is preferred, levofloxacin is another alternative that can be administered orally, but all studies except one that used levofloxacin containing triple therapy were from China. The only study outside of China showed that dual therapy was not inferior to levofloxacin containing triple therapy. Ofloxacin in combination with rifampicin is another fully orally administrable alternative combination with equal efficacy to the doxycycline + rifampicin. However, replacing either doxycycline or rifampicin with ciprofloxacin is not recommended due to higher risks of treatment failure or relapses. Though aminoglycosides such as gentamycin and amikacin have been used in combination with doxycycline effectively in the treatment of brucellosis, they also require parenteral administration. Yet, unlike streptomycin, aminoglycosides have been used for a shorter duration of 5–7 days. Overall, for uncomplicated brucellosis, there are many effective, safe, and relatively cheap treatment options including oral only antibiotic combinations. For complicated and focal diseases adding an aminoglycoside, levofloxacin or streptomycin as the third antibiotic or as a replacement should be discussed with the patient highlighting the changes to treatment duration, mode of administration and side effects. Overall, severe adverse events were uncommon among the studies included in this review, but two out of three studies that had a patient group treated with cotrimoxazole reported serious adverse events in the cotrimoxazole group (the third study did not mention adverse events).

A recently published systematic review which summarized evidence from 51 studies originating from 10 Middle Eastern and North African countries on antibiotic resistance of *Brucella melitensis and B. abortus* isolates (of human and animal origin) confirmed susceptibility to most commonly used antibiotics to treat human brucellosis^53^. However, varying techniques used in laboratories to assess the minimum inhibitory concentration (MIC) of pathogenic *Brucella spp*.^54^ as well as the slow growing nature of bacteria pose challenges in accurate determination of MIC^53^. Therefore, antibiotic sensitivity patterns reported in the laboratory may not always guarantee clinical cure in patients. Also, given the risk of laboratory acquired infections, antibiotic sensitivity testing may not be routinely performed as well.^55^ Without it, the treating clinicians can only depend on their subjective observations to define clinical improvement and hence a cure. This makes it even more difficult to differentiate a treatment failure from a relapse if symptoms reappear. These real-life difficulties affect the accuracy of numbers reported for antibiotic sensitivity, treatment success and relapses in clinical trials.

Regarding the risk of bias most trials had a high risk of performance and detection bias as they were open label studies. Given the longer duration of therapy it may be difficult to mask the antibiotic regimens due to logistical reasons. This is more so when parenteral preparations are used. All trials except eight randomly allocated participants to treatment arms but many did not do allocation concealment (or did not mention it in the publication). We used an arbitrary cut-off of 10% attrition to define attrition bias up to the point of completion of follow up. On this criterion nearly a quarter of the studies had a high attrition bias (9/34, 27%). However, each study had different durations of follow up for relapses and those that followed up patients for longer had a higher risk of attrition. Many Chinese studies described the initial treatment response in three categories (cured, improved with treatment, failure) and did not report on relapses (therefore were noted as having high reporting or other biases). For the outcome of treatment failures, patients not explicitly defined as such were considered as cured (including the “improved with treatment” category), but admittedly this intermediate category is a source of bias.

For some of the meta-analyses results, the certainty of evidence was graded as “moderate” due to confirmation of effect in the same direction in multiple studies (consistency), and after consideration of risks of bias in individual studies. Except for one comparison, none were classified as high certainty evidence due to indirectness (e.g. triple therapy reserved for severe disease, trials from a limited number of countries, mostly Iran, China and Turkey) and imprecision (small number of events, small sample sizes).

The last comprehensive meta-analysis which compared all combinations of antibiotic therapies for brucellosis was published in 2012 (del Pozo et al.) but our review includes 17 new studies published after 2012, including those published in languages other than English. Unlike the previous review, we imposed a time limit to our search (25 years prior to search date) to capture the most recent evidence as patterns of antibiotic resistance change over time. The 2012 review included 15 studies that were published before the time limit cut-off of this study. Despite this, their findings were similar to ours with regard to the superiority of doxycycline + streptomycin over doxycycline + rifampicin for relapse prevention, though their evidence was drawn from a larger number of studies. They also found that ofloxacin and rifampicin was equally effective to doxycycline and rifampicin like in our analysis. Del Pozo et al. also compared doxycycline + streptomycin vs. doxycycline + gentamycin and found equal efficacy (2 studies), a finding similar to ours. Other meta-analyses done in the previous study had lumped all trials that administered a quinolone antibiotic together in comparisons, an approach we did not adapt as the efficacy and pharmacokinetics of each quinolone is different. Del Pozo et al. also did not compare the efficacy of triple therapy vs. dual therapy. A later systematic review^15^ which exclusively looked at this comparison concluded that triple therapy is superior, inline with our results.

Regarding the limitations of this review, the search was limited to the databases mentioned in the methods section. Non-peer reviewed publications and preprints were excluded. When an article was published in a language other than English, we made every effort to translate and include it. On this basis several full-text articles published in Persian, Mandarin and Spanish were considered during the full-text screening. Despite this the available evidence is mostly limited to few endemic countries in the Middle East, Far East Asia and in the Mediterranean region. Though these countries collectively may share a larger proportion of the global burden of brucellosis due to limited livestock vaccination and less stringent occupational safety measures in at-risk industries and services^56,57^, epidemiological data are also lacking from many other low- and middle-income countries resulting in an underestimation of true global disease burden. We only included published data, and this limited the ability to do sensitivity analyses for adults and children given that age cut-offs to define children were different in each country. Also, the duration of follow up to detect relapses varied between studies and all studies reported on the number of relapses at the end of the follow up rather than the time to each relapse. This prevented a time-to event analysis which would have been more informative in determining the efficacy of antibiotics for relapse prevention.

## Conclusions

The oral only and commonly used doxycycline (100 mg twice daily for 6–8 weeks) + rifampicin (600–900 mg/d for 6–8 weeks) antibiotic combination was equally effective or superior to any other orally administered antibiotic combination assessed for both initial cure and relapse prevention in human brucellosis. It was only inferior to the triple combinations of doxycycline + rifampicin + streptomycin (750–1000 mg per day for 1–3 weeks) or doxycycline + rifampicin + levofloxacin (500 mg daily orally for 6 weeks). The doses and durations mentioned are the typical doses used in most studies (Supplementary Table 2). Available data is limited to small sized studies from only seven countries, and in future these conclusions may change as more evidence from larger randomized controlled trials conducted in other countries become available.

### Supplementary Information

Supplementary Information.

## Data Availability

The minimum dataset for this paper is available within the manuscript and its supplementary material. This review only relied on published data already publicly available.
