# Supplementary material for: A systematic review and meta-analysis of comparative clinical studies on antibiotic treatment of brucellosis
Source: Sci Rep. 2024 Aug 16;14:19037. doi: 10.1038/s41598-024-69669-w (PMC11329684; doi:10.1038/s41598-024-69669-w)
Supplement: Supplementary file 1 — Supplementary Information. [file 41598_2024_69669_MOESM1_ESM.pdf]

PRISMA 2020 for Abstracts Checklist

Section and Topic	Item #	Checklist item	Reported (Yes/No)
**TITLE**			
Title	1	Identify the report as a systematic review.	Yes
**BACKGROUND**			
Objectives	2	Provide an explicit statement of the main objective(s) or question(s) the review addresses.	Yes
**METHODS**			
Eligibility criteria	3	Specify the inclusion and exclusion criteria for the review.	Yes
Information sources	4	Specify the information sources (e.g. databases, registers) used to identify studies and the date when each was last searched.	Yes
Risk of bias	5	Specify the methods used to assess risk of bias in the included studies.	Yes
Synthesis of results	6	Specify the methods used to present and synthesise results.	Yes
**RESULTS**			
Included studies	7	Give the total number of included studies and participants and summarise relevant characteristics of studies.	Yes
Synthesis of results	8	Present results for main outcomes, preferably indicating the number of included studies and participants for each. If meta-analysis was done, report the summary estimate and confidence/credible interval. If comparing groups, indicate the direction of the effect (i.e. which group is favoured).	Yes
**DISCUSSION**			
Limitations of evidence	9	Provide a brief summary of the limitations of the evidence included in the review (e.g. study risk of bias, inconsistency and imprecision).	In main manuscript*
Interpretation	10	Provide a general interpretation of the results and important implications.	Yes
**OTHER**			
Funding	11	Specify the primary source of funding for the review.	Submission system
Registration	12	Provide the register name and registration number.	In main manuscript*

**Due to word limits for the abstract this information is presented in main manuscript*

From: Page MJ, McKenzie JE, Bossuyt PM, Boutron I, Hoffmann TC, Mulrow CD, et al. The PRISMA 2020 statement: an updated guideline for reporting systematic reviews. BMJ 2021;372:n71. doi: 10.1136/bmj.n71

For more information, visit: http://www.prisma-statement.org/

PRISMA 2020 Checklist

Section and Topic	Item #	Checklist item	Details of where the item is reported
**TITLE**			
Title	1	Identify the report as a systematic review.	1
**ABSTRACT**			
Abstract	2	See the PRISMA 2020 for Abstracts checklist.	Completed
**INTRODUCTION**			
Rationale	3	Describe the rationale for the review in the context of existing knowledge.	Page (P) 4, Paragraph (Pa) 1
Objectives	4	Provide an explicit statement of the objective(s) or question(s) the review addresses.	P5 Pa1 (The aim of…)
**METHODS**			
Eligibility criteria	5	Specify the inclusion and exclusion criteria for the review and how studies were grouped for the syntheses.	P5 Pa2 (Under subheading of study eligibility)
Information sources	6	Specify all databases, registers, websites, organisations, reference lists and other sources searched or consulted to identify studies. Specify the date when each source was last searched or consulted.	P6 Pa1 (Under subheading search strategy)
Search strategy	7	Present the full search strategies for all databases, registers and websites, including any filters and limits used.	P6 Pa1 (as above) and Supplementary Tabe 1
Selection process	8	Specify the methods used to decide whether a study met the inclusion criteria of the review, including how many reviewers screened each record and each report retrieved, whether they worked independently, and if applicable, details of automation tools used in the process.	P6 Pa2 (Under subheading data extraction and analysis)
Data collection process	9	Specify the methods used to collect data from reports, including how many reviewers collected data from each report, whether they worked independently, any processes for obtaining or confirming data from study investigators, and if applicable, details of automation tools used in the process.	P6 Pa2 (Under subheading data extraction and analysis)
Data items	10a	List and define all outcomes for which data were sought. Specify whether all results that were compatible with each outcome domain in each study were sought (e.g. for all measures, time points, analyses), and if not, the methods used to decide which results to collect.	P5 Pa 2 (Outcomes assessed were..), Supplementary Table 2 – Characteristics of included studies
	10b	List and define all other variables for which data were sought (e.g. participant and intervention characteristics, funding sources). Describe any assumptions made about any missing or unclear information.	P6 Pa2 (From the eligible studies…)
Study risk of bias assessment	11	Specify the methods used to assess risk of bias in the included studies, including details of the tool(s) used, how many reviewers assessed each study and whether they worked independently, and if applicable, details of automation tools used in the process.	P7 Pa 1 (under subheading risk of bias and certainty of evidence)

PRISMA 2020 Checklist

Section and Topic	Item #	Checklist item	Details of where the item is reported
Effect measures	12	Specify for each outcome the effect measure(s) (e.g. risk ratio, mean difference) used in the synthesis or presentation of results.	P6 Pa 2 (The effect size of each treatment comparison…)
Synthesis methods	13a	Describe the processes used to decide which studies were eligible for each synthesis (e.g. tabulating the study intervention characteristics and comparing against the planned groups for each synthesis (item #5)).	P5 Pa 2 (If two or more similar studies were available…)
	13b	Describe any methods required to prepare the data for presentation or synthesis, such as handling of missing summary statistics, or data conversions.	P6 Pa 2 (All meta-analyses were initially…)
	13c	Describe any methods used to tabulate or visually display results of individual studies and syntheses.	P6 Pa 2 (All meta-analyses were visually displayed with Forest plots)
	13d	Describe any methods used to synthesize results and provide a rationale for the choice(s). If meta-analysis was performed, describe the model(s), method(s) to identify the presence and extent of statistical heterogeneity, and software package(s) used.	P6 Pa 2 (Under subheading Data extraction and analysis)
	13e	Describe any methods used to explore possible causes of heterogeneity among study results (e.g. subgroup analysis, meta-regression).	P6 Pa 2 (The heterogeneity was assessed…)
	13f	Describe any sensitivity analyses conducted to assess robustness of the synthesized results.	P6 Pa 2 (All meta-analyses were initially…)
Reporting bias assessment	14	Describe any methods used to assess risk of bias due to missing results in a synthesis (arising from reporting biases).	P6 Pa 2 (All meta-analyses were initially…)
Certainty assessment	15	Describe any methods used to assess certainty (or confidence) in the body of evidence for an outcome.	P7 Pa 1 (under subheading risk of bias and certainty of evidence)
**RESULTS**			
Study selection	16a	Describe the results of the search and selection process, from the number of records identified in the search to the number of studies included in the review, ideally using a flow diagram.	P7 Pa 2 (Thirty four studies published …)
	16b	Cite studies that might appear to meet the inclusion criteria, but which were excluded, and explain why they were excluded.	Supplementary Table 3
Study characteristics	17	Cite each included study and present its characteristics.	P7 Pa 2 and Supplementary Table 2

PRISMA 2020 Checklist

Section and Topic	Item #	Checklist item	Details of where the item is reported
Risk of bias in studies	18	Present assessments of risk of bias for each included study.	P8 Pa3 (under subheading Risk of Bias), Figure 2
Results of individual studies	19	For all outcomes, present, for each study: (a) summary statistics for each group (where appropriate) and (b) an effect estimate and its precision (e.g. confidence/credible interval), ideally using structured tables or plots.	Tables 1 and 2
Results of syntheses	20a	For each synthesis, briefly summarise the characteristics and risk of bias among contributing studies.	P8 Pa 3 under subheading Risk of Bias), Figure 2
	20b	Present results of all statistical syntheses conducted. If meta-analysis was done, present for each the summary estimate and its precision (e.g. confidence/credible interval) and measures of statistical heterogeneity. If comparing groups, describe the direction of the effect.	P9 (under subheading “Risk of Treatment Failure”), P13 (Under subheading “Risk of Relapse”), Figures 3 -7, Supplementary figures
	20c	Present results of all investigations of possible causes of heterogeneity among study results.	As above
	20d	Present results of all sensitivity analyses conducted to assess the robustness of the synthesized results.	P17 (Under subheading “Sensitivity analysis”)
Reporting biases	21	Present assessments of risk of bias due to missing results (arising from reporting biases) for each synthesis assessed.	Supplementary figures, P17 (Under subheading “Sensitivity analysis”)
Certainty of evidence	22	Present assessments of certainty (or confidence) in the body of evidence for each outcome assessed.	P9 (under subheading “Risk of Treatment Failure”) and P13 (Under subheading “Risk of Relapse”)
**DISCUSSION**			
Discussion	23a	Provide a general interpretation of the results in the context of other evidence.	P18 Pa1
	23b	Discuss any limitations of the evidence included in the review.	P19 Pa2 (Regarding risk of bias…), P20 Pa 2 (“Regarding the

Section and Topic	Item #	Checklist item	Details of where the item is reported
			limitations…”)
	23c	Discuss any limitations of the review processes used.	P19 Pa2 (Regarding risk of bias…), P20 Pa 2 (“Regarding the limitations…”)
	23d	Discuss implications of the results for practice, policy, and future research.	P20 (Under “Conclusions”)
**OTHER INFORMATION**			
Registration and protocol	24a	Provide registration information for the review, including register name and registration number, or state that the review was not registered.	P7 (“The protocol for …”)
	24b	Indicate where the review protocol can be accessed, or state that a protocol was not prepared.	P7 – Protocol registered in PROSPERO
	24c	Describe and explain any amendments to information provided at registration or in the protocol.	Not applicable
Support	25	Describe sources of financial or non-financial support for the review, and the role of the funders or sponsors in the review.	Under subheading “Funding” or information provided in submission system
Competing interests	26	Declare any competing interests of review authors.	Under subheading “Competing interests” or information provided in submission system
Availability of data, code and other materials	27	Report which of the following are publicly available and where they can be found: template data collection forms; data extracted from included studies; data used for all analyses; analytic code; any other materials used in the review.	Under subheading “Data availability” or information provided in submission system

From: Page MJ, McKenzie JE, Bossuyt PM, Boutron I, Hoffmann TC, Mulrow CD, et al. The PRISMA 2020 statement: an updated guideline for reporting systematic reviews. BMJ 2021;372:n71. doi: 10.1136/bmj.n71

For more information, visit: http://www.prisma-statement.org/

Supplementary Figures

(D – Doxycycline, R – Rifampicin, S – Streptomycin, Co – Cotrimoxazole, G – Gentamycin, A – Amikacin, L – Levofloxacin, O – Ofloxacin, C – Ciprofloxacin)

SF1. Comparison – D + R + S vs. D + R, Outcome – Treatment failure, Analysis – Per protocol (Figures for intention to treat analysis are included in main manuscript)

	D + R		D + R + S			Risk Ratio
**Study or Subgroup**	**Events**	**Total**	**Events**	**Total**	**Weight**	**M-H, Fixed, 95% CI**
Bayindir, 2003	3	20	0	22	3.8%	7.67 [0.42, 139.83]
El-Miedany, 2003	15	32	20	75	96.2%	1.76 [1.04, 2.98]
**Total (95% CI)**		**52**		**97**	**100.0%**	**1.98 [1.17, 3.35]**
Total events	18		20			

Heterogeneity: Chi² = 1.04, df = 1 (P = 0.31); I² = 4%
Test for overall effect: Z = 2.56 (P = 0.01)

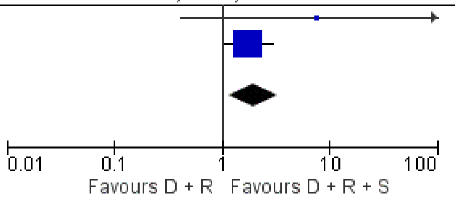

SF2. Comparison – D + R + S vs. D + R, Outcome – Relapse, Analysis – Per protocol (Figures for intention to treat analysis are included in main manuscript)

	D + R		D + R + S			Risk Ratio
**Study or Subgroup**	**Events**	**Total**	**Events**	**Total**	**Weight**	**M-H, Fixed, 95% CI**
Bayindir, 2003	2	20	0	22	66.2%	5.48 [0.28, 107.62]
El-Miedany, 2003	10	17	0	55	33.8%	65.33 [4.03, 1060.39]
**Total (95% CI)**		**37**		**77**	**100.0%**	**25.68 [4.44, 148.60]**
Total events	12		0			

Heterogeneity: Chi² = 1.47, df = 1 (P = 0.23); I² = 32%
Test for overall effect: Z = 3.62 (P = 0.0003)

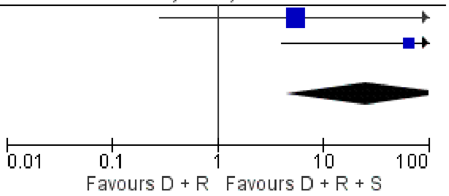

SF3. Comparison – D + R vs. C + R, Outcome – Treatment failure, Analysis – Intention to treat

	D + R		C + R			Risk Ratio
**Study or Subgroup**	**Events**	**Total**	**Events**	**Total**	**Weight**	**M-H, Fixed, 95% CI**
Agalar 1999	0	20	0	20		Not estimable
Keramat 2009	2	61	3	62	57.9%	0.68 [0.12, 3.91]
Taghavee 2011	1	27	2	23	42.1%	0.43 [0.04, 4.40]
**Total (95% CI)**		**108**		**105**	**100.0%**	**0.57 [0.14, 2.30]**
Total events	3		5			

Heterogeneity: Chi² = 0.10, df = 1 (P = 0.76); I² = 0%
Test for overall effect: Z = 0.79 (P = 0.43)

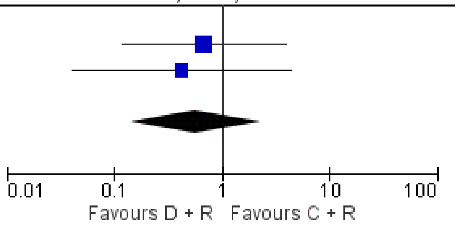

SF4. Comparison – D + R vs. C + R, Outcome – Relapse, Analysis – Per protocol (Figures for intention to treat analysis are included in main manuscript)

	D + R		C + R			Risk Ratio
**Study or Subgroup**	**Events**	**Total**	**Events**	**Total**	**Weight**	**M-H, Fixed, 95% CI**
Agalar 1999	2	10	3	15	19.0%	1.00 [0.20, 4.95]
Keramat 2009	2	26	3	36	19.9%	0.92 [0.17, 5.14]
Taghavee 2011	1	26	7	21	61.2%	0.12 [0.02, 0.87]
**Total (95% CI)**		**62**		**72**	**100.0%**	**0.44 [0.18, 1.11]**
Total events	5		13			

Heterogeneity: Chi² = 3.41, df = 2 (P = 0.18); I² = 41%
Test for overall effect: Z = 1.73 (P = 0.08)

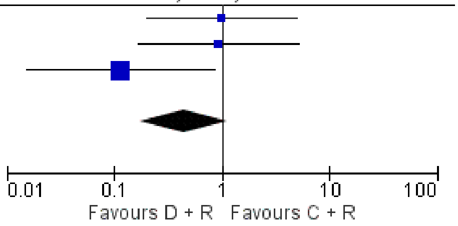

SF5. Comparison – D + R vs. O + R, Outcome – Treatment failure, Analysis – Intention to treat

	D + R		O + R			Risk Ratio
**Study or Subgroup**	**Events**	**Total**	**Events**	**Total**	**Weight**	**M-H, Fixed, 95% CI**
Bayindir, 2003	3	20	5	19	50.4%	0.57 [0.16, 2.06]
Ersoy, 2005	1	45	1	41	10.3%	0.91 [0.06, 14.10]
Hashemi, 2012	10	73	4	73	39.3%	2.50 [0.82, 7.61]
**Total (95% CI)**		**138**		**133**	**100.0%**	**1.36 [0.64, 2.92]**
Total events	14		10			

Heterogeneity: Chi² = 2.99, df = 2 (P = 0.22); I² = 33%
Test for overall effect: Z = 0.80 (P = 0.42)

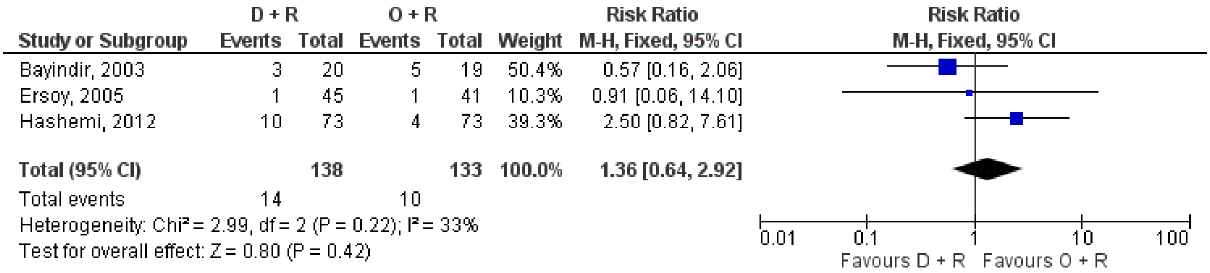

SF6. Comparison – D + R vs. D + S, Outcome – Treatment failure, Analysis – Intention to treat

	D + R		D + S			Risk Ratio
**Study or Subgroup**	**Events**	**Total**	**Events**	**Total**	**Weight**	**M-H, Fixed, 95% CI**
Bayindir, 2003	3	20	4	21	48.3%	0.79 [0.20, 3.09]
Ersoy, 2005	1	45	1	32	14.5%	0.71 [0.05, 10.95]
Hashemi, 2012	10	73	3	73	37.2%	3.33 [0.96, 11.62]
**Total (95% CI)**		**138**		**126**	**100.0%**	**1.72 [0.75, 3.93]**
Total events	14		8			

Heterogeneity: Chi² = 2.74, df = 2 (P = 0.25); I² = 27%
Test for overall effect: Z = 1.29 (P = 0.20)

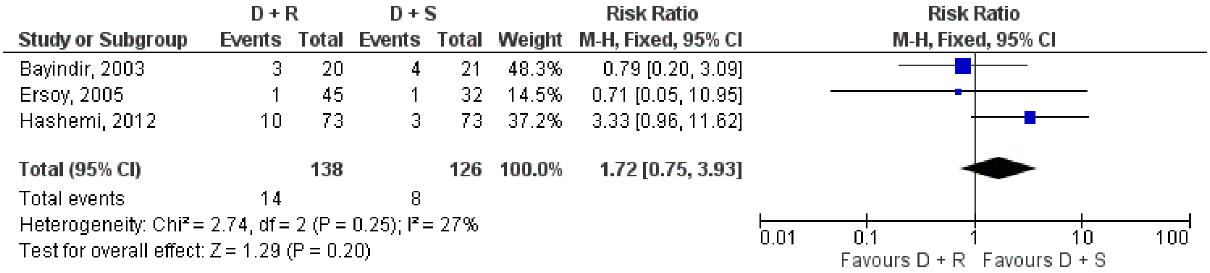

SF7. Comparison – D + R vs. D + S, Outcome – Relapse, Analysis – Intention to treat

	D + R		D + S			Risk Ratio
**Study or Subgroup**	**Events**	**Total**	**Events**	**Total**	**Weight**	**M-H, Fixed, 95% CI**
Bayindir, 2003	2	20	0	21	7.0%	5.24 [0.27, 102.81]
Ersoy, 2005	6	45	3	32	50.1%	1.42 [0.38, 5.27]
Hashemi, 2012	9	73	3	73	42.9%	3.00 [0.85, 10.64]
**Total (95% CI)**		**138**		**126**	**100.0%**	**2.37 [1.01, 5.55]**
Total events	17		6			

Heterogeneity: Chi² = 0.99, df = 2 (P = 0.61); I² = 0%
Test for overall effect: Z = 1.98 (P = 0.05)

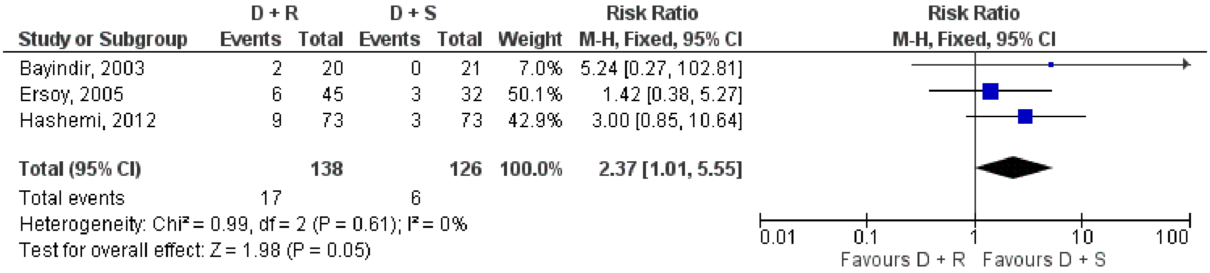

SF8. Comparison – D + R vs. D + S, Outcome – Relapse, Analysis – Per protocol

	D + R		D + S			Risk Ratio
**Study or Subgroup**	**Events**	**Total**	**Events**	**Total**	**Weight**	**M-H, Fixed, 95% CI**
Bayindir, 2003	2	20	0	21	7.1%	5.24 [0.27, 102.81]
Ersoy, 2005	6	45	3	32	51.2%	1.42 [0.38, 5.27]
Hashemi, 2012	9	59	3	65	41.7%	3.31 [0.94, 11.63]
**Total (95% CI)**		**124**		**118**	**100.0%**	**2.48 [1.06, 5.79]**
Total events	17		6			

Heterogeneity: Chi² = 1.13, df = 2 (P = 0.57); I² = 0%
Test for overall effect: Z = 2.10 (P = 0.04)

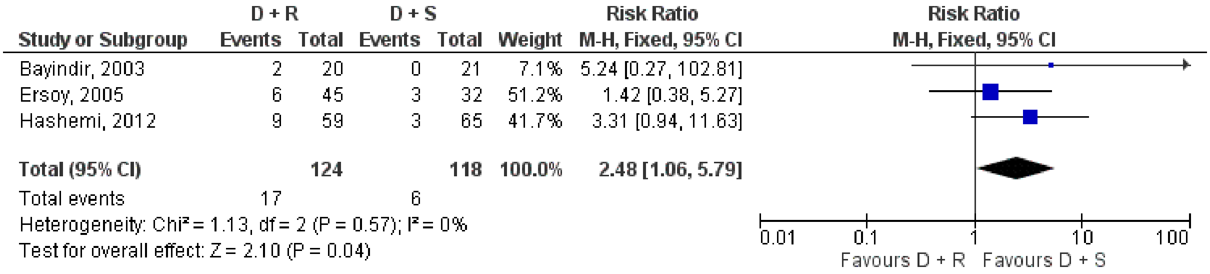

SF9. Comparison – O + R vs. D + S, Outcome – Treatment failure, Analysis – Intention to treat

	O + R		D + S			Risk Ratio
**Study or Subgroup**	**Events**	**Total**	**Events**	**Total**	**Weight**	**M-H, Fixed, 95% CI**
Bayindir, 2003	5	19	4	21	48.0%	1.38 [0.43, 4.40]
Ersoy, 2005	1	41	1	32	14.2%	0.78 [0.05, 12.00]
Hashemi, 2012	4	73	3	73	37.9%	1.33 [0.31, 5.75]
**Total (95% CI)**		**133**		**126**	**100.0%**	**1.28 [0.54, 3.03]**
Total events	10		8			

Heterogeneity: Chi² = 0.15, df = 2 (P = 0.93); I² = 0%
Test for overall effect: Z = 0.56 (P = 0.58)

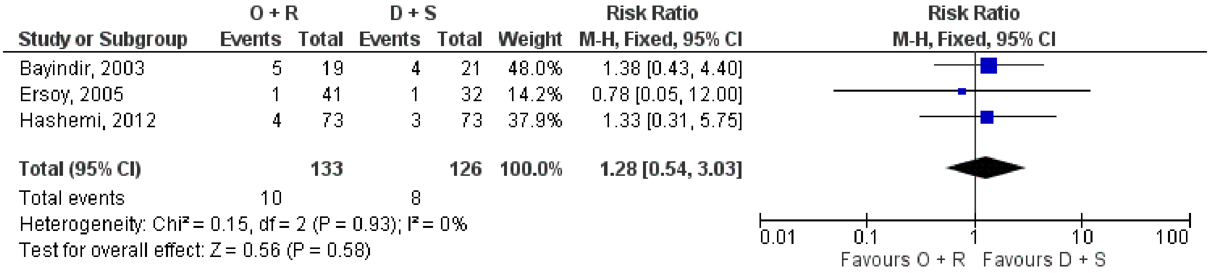

SF10. Comparison – O + R vs. D + S, Outcome – Relapse, Analysis – Intention to treat

Study or Subgroup	O + R Events	O + R Total	D + S Events	D + S Total	Weight	Risk Ratio M-H, Fixed, 95% CI
Bayindir, 2003	5	19	0	21	7.0%	12.10 [0.71, 205.25]
Ersoy, 2005	5	41	3	32	49.2%	1.30 [0.34, 5.04]
Hashemi, 2012	5	73	3	73	43.8%	1.67 [0.41, 6.72]
**Total (95% CI)**		**133**		**126**	**100.0%**	**2.21 [0.93, 5.27]**
Total events	15		6			

Heterogeneity: Chi² = 2.13, df = 2 (P = 0.34); I² = 6%
Test for overall effect: Z = 1.79 (P = 0.07)

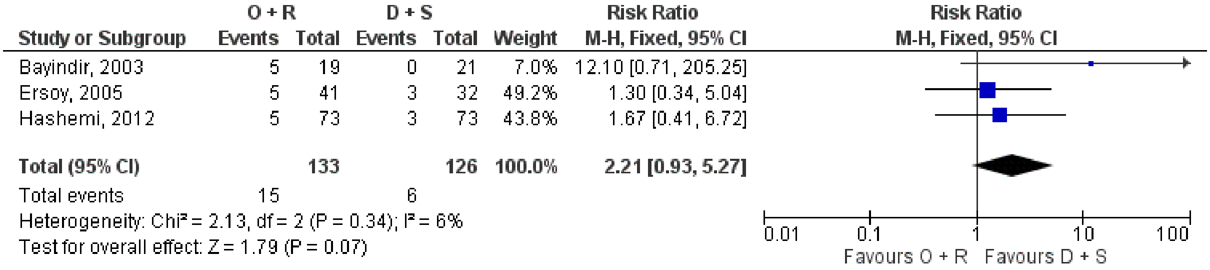

SF11. Comparison – D + R vs. D + C, Outcome – Treatment failure, Analysis – per protocol (Figures for intention to treat analysis are included in main manuscript)

Study or Subgroup	D + R Events	D + R Total	D + C Events	D + C Total	Weight	Risk Ratio M-H, Fixed, 95% CI
Didgar 2012	3	41	7	39	49.4%	0.41 [0.11, 1.47]
Keramat 2009	2	61	7	55	50.6%	0.26 [0.06, 1.19]
**Total (95% CI)**		**102**		**94**	**100.0%**	**0.33 [0.12, 0.88]**
Total events	5		14			

Heterogeneity: Chi² = 0.20, df = 1 (P = 0.65); I² = 0%
Test for overall effect: Z = 2.21 (P = 0.03)

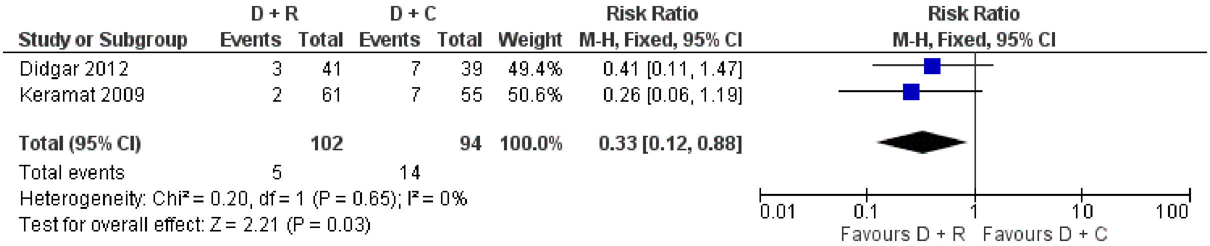

SF12. Comparison – D + S vs. D + G, Outcome – Treatment failure, Analysis – Intention to treat

Study or Subgroup	D + S Events	D + S Total	D + G Events	D + G Total	Weight	Risk Ratio M-H, Fixed, 95% CI
Roushan 2010	4	82	2	82	50.0%	2.00 [0.38, 10.62]
Roushan, 2006	4	100	2	100	50.0%	2.00 [0.37, 10.67]
**Total (95% CI)**		**182**		**182**	**100.0%**	**2.00 [0.61, 6.52]**
Total events	8		4			

Heterogeneity: Chi² = 0.00, df = 1 (P = 1.00); I² = 0%
Test for overall effect: Z = 1.15 (P = 0.25)

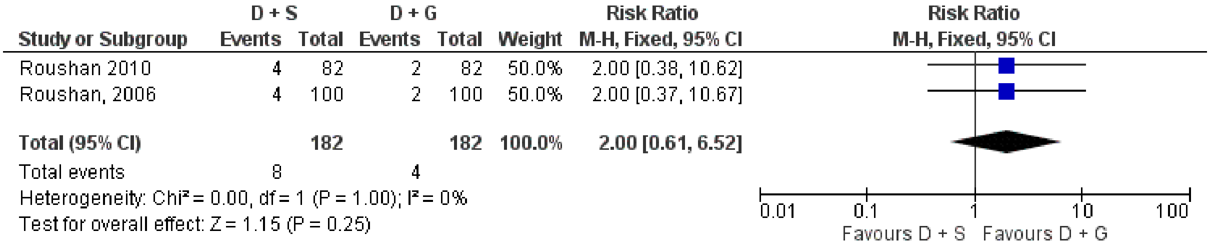

SF13. Comparison – D + S vs. D + G, Outcome – Relapse, Analysis – Intention to treat

Study or Subgroup	D + S Events	D + S Total	D + G Events	D + G Total	Weight	Risk Ratio M-H, Fixed, 95% CI
Roushan 2010	5	82	2	82	40.0%	2.50 [0.50, 12.52]
Roushan, 2006	3	100	3	100	60.0%	1.00 [0.21, 4.84]
**Total (95% CI)**		**182**		**182**	**100.0%**	**1.60 [0.53, 4.80]**
Total events	8		5			

Heterogeneity: Chi² = 0.64, df = 1 (P = 0.43); I² = 0%
Test for overall effect: Z = 0.84 (P = 0.40)

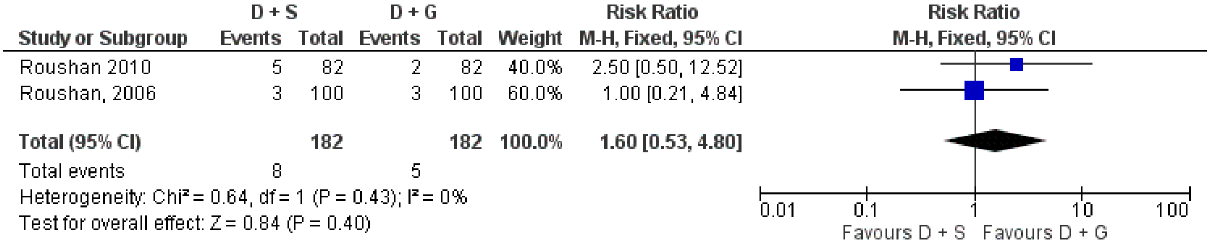

SF14. Comparison – D + R vs. D Outcome – Treatment failure, Intention to treat.

Study or Subgroup	D + R Events	D + R Total	D Events	D Total	Weight	Odds Ratio M-H, Fixed, 95% CI
Feng 2016	3	50	11	49	64.2%	0.22 [0.06, 0.85]
Liu 2018	1	36	6	36	35.8%	0.14 [0.02, 1.25]
**Total (95% CI)**		**86**		**85**	**100.0%**	**0.19 [0.06, 0.60]**
Total events	4		17			

Heterogeneity: Chi² = 0.11, df = 1 (P = 0.74); I² = 0%
Test for overall effect: Z = 2.83 (P = 0.005)

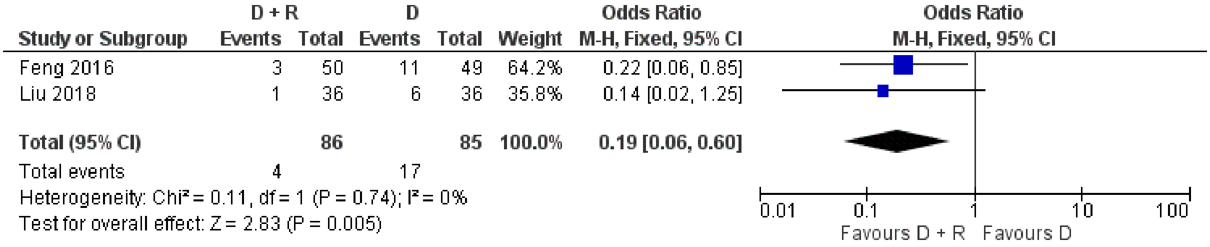

Supplementary Tables

Supplementary Table 1. Search strategy and results (last day of search – 06/06/2023)

Database	Search strategy	Limits	Number of hits
PUBMED	(brucellosis OR brucella) AND (treat* OR intervention) AND (prospective OR random* OR control* OR trial OR cohort)	Title and abstract	644
Scopus	Same as above	Title, abstract and keywords	1726
Web of Science	Same as above	Title, abstract and keywords	762
CINAHL	Same as above	Title, abstract and keywords	740
EMBASE	Same as above	Title, abstract and keyword headings (kw)	1142
China Academic Journals (CKNI)*	Same as above translated to Mandarin	Title, abstract and keywords	173

*Last date of search was 12/03/2024 for this resource

Supplementary Table 2. Characteristics of Included Studies

**Study and setting**	**Design**	**Participants**	**Intervention and comparators***	**Outcome definitions**	**Diagnosis of Brucellosis**
Agalar et al., 1999, Turkey[1]	Open, randomized, clinical trial	Patients aged > 15 years	D 100mg bd + R 600mg/d for 45 days vs. C 1g /d vs. R 600mg/d for 30 d	Failure: not defined<br>Relapse: Reappearance of symptoms / signs of the disease and positive serologic tests during 12-months after treatment was stopped.	Clinical features + Positive blood culture
Alavi et al., 2007, Iran[2]	Randomized, clinical trial	Patients aged > 15 years	D 100mg bd + R 600mg /d for 8 weeks vs. D 100mg bd vs. Cotrimoxazole 1920mg/d for 8 weeks	Failure: Persistent symptoms and increase in titres after an initial decline during treatment<br>Relapse: Reappearance of symptoms / signs of the disease and increasing titres by 2-ME (IgG) and wright tests within 6-months after treatment was stopped.	>1/ 80 standard tube agglutination titer (STAT) of antibodies to brucella (Wright) with a 2-mercaptoethanol (2 ME) >1/40, in association with compatible Clinical findings
AliKhani et al., 2007, Iran[3]	Single-blind randomized trial	Patients aged > 15 years	D 100mg bd + S 750-1000mg daily in first month, followed by D 100mg bd + R 600mg /d in 2nd and 3rd months vs. O 400mg/d + S 750-1000mg/d in first month, R 600mg/d + O 200mg/ d for 2nd and 3rd months	Failure: Persistence of symptoms and no decline in 2ME and Wright titers<br>Relapse: Recurrence of clinical symptoms accompanied by increase in 2ME (IgG) and Wright titers (equal to or more than 1/160 and 1/20 respectively)	Clinical features + Wright and 2ME tests with titers equal to or more than 1/160 and 1/20

				during the follow-up period	
Alp et al., 2006, Turkey[4]	Open, non-randomized trial	Patients aged > 16 years	D 100mg bd for a minimum of 12 weeks + IM S 1g/d for 21d vs. C 1g /d + R 600mg/d for a minimum of 12 weeks	Failure: Symptoms and signs of the disease persisted or increased, with worsening MRI findings, at the end of 12 weeks therapy<br>Relapse: Reappearance of symptoms or signs of the disease or new positive blood cultures during the 12 months after therapy	Spinal brucellosis only - MRI evidence + Positive wright test with a titre > 1/160, and/or positive culture of brucella
Bayindir et al., 2003, Turkey[5]	Prospective randomized trial	Patients aged > 21 years	S 1 g/day IM for 15 d and tetracycline-HCl, 500 mg every 6 h orally for 45 d vs. S 1 g/day IM for 15 d and D 100 mg bd vs. D 100 mg bd for 45 d and R 15 mg/kg per day for 45 d vs. O, 200 mg bd for 45 d and R 15 mg/kg per day for 45 d vs. S 1 g/day IM for 15 d and D 100 mg bd for 45 d plus R 15 mg/kg per day for 45 d	Failure: Continued clinical and bacterial/serological evidence of infection after 45 days<br>Relapse: Recurrence of clinical signs and > 4-fold rise in SAT IgG with 2-ME	Clinical features + >1/160 titre on STAT or Rose Bengal test and / or positive culture
Didgar et al., 2012, Iran[6]	Triple-blinded randomized trial	Patients aged >17 years	D 100mg bd + C 1g /d for a minimum of 12 weeks vs. D 100mg bd + R 300mg bd for a minimum of 12 weeks	Failure: Persistence of clinical symptoms or persistently high antibody titres after 12 weeks of treatment<br>Relapse: Reappearance	Clinical features + Wright and 2ME tests with titers equal to or more than 1/160 and 1/80 and / or positive culture

				of symptoms or increase in 2ME and Wright titers (IgG)	
El-Miedany et al., 2003, Saudi Arabia[7]	Prospective observational study	Patients aged > 21 years	R 900 mg/day + D, 100 mg bd vs. R 900 mg/day + cotrimoxazole, 960 mg bd vs. IM S 1 g/day for 14 d + R 900 mg/d + D 100 mg bd	Failure: Not reaching STAT ≤ 1:80, and IgG / IgM (by ELISA) antibody titers not reaching < 50 U/ml<br>Relapse: Recurrence of clinical signs (clinical relapse), in addition to either new positive blood culture (bacterial relapse) and/or a 4-fold increase in serological titre (IgG by agglutination tests) after completion of therapy	Clinical features + >1/160 titre on STA and / or positive culture
Ersoy et al., 2005, Turkey[8]	Randomized unblinded trial	Patients aged >16 years	O 400 mg/day + R 600 mg/day for 6 weeks, vs. D 100mg bd + R 600 mg/day for 6 weeks vs. D 100 mg bd for 6 weeks + IM S 1 g, for 3 weeks	Failure: not defined<br>Relapse: Recurrence of clinical symptoms with rising antibody titre or positive culture	Clinical features + >1/160 titre on STAT and / or positive culture
Feng et al. 2016, China[9]	Randomized controlled trial of patients with brucella osteoarthritis	Not mentioned	D 200mg/d for 8 weeks vs. D 200mg/d + R 450mg/d for 8 weeks	Failure: Continued symptoms and positive serological tests<br>Relapse: Not defined	Clinical features + China CDC Diagnostic criteria[10]
Hasanain et al., 2016, Egypt[11]	Randomized unblinded trial	Adult patients	D 100mg bd + R 900 mg/day, for six weeks vs. D 100mg bd, R 900 mg/day, and L 500 mg/day, for six weeks	Failure: Persistence of clinical symptoms/signs after 6 weeks of treatment<br>Relapse: Recurrence of	Clinical features + >1/160 titre on STAT + exposure to risk factors

				the clinical manifestations with a single positive antibody titer within six months after ending therapy	
Roushan et al., 2006, Iran[12]	Prospective randomized trial	Adult patients	IM S 1g for 14 d + D 100mg bd for 45 d vs. 5 mg/kg per day of gentamicin for 7 d + D 100mg bd for 45 d	Failure: persistence of clinical symptoms of disease after completion of treatment or discontinuation of treatment due to serious adverse effects associated with >1 of the drugs<br>Relapse: Clinical symptoms and signs of brucellosis reappeared and a previously reduced titre of STAT or 2ME (IgG) increased after completion of therapy	STA titer >1:320 and 2-Mercaptoethanol (2ME) titer >1:80 in those who had clinical findings compatible with Brucellosis.
Hashemi et al., 2012, Iran[13]	Prospective randomized trial	Patients aged > 17 years	D 100mg bd for 6 weeks + S 1g daily for 21d vs. D 100mg bd + R 15 mg/kg daily for 6 weeks vs. O 800 mg daily + R 15 mg/kg daily for 6 weeks	Failure: persistence of symptoms and signs at the end of 6 weeks of therapy<br>Relapse: Reappearance of symptoms and signs accompanied by a 2-ME titer >1/80 (IgG) during the follow-up period	Clinical presentation + the presence of significant titers of specific antibodies (standard tube agglutination >1/160, Coombs test >1/160, 2-mercaptoetanol (2-ME) >1-80, or Brucella IgG-ELISA positive) and/or a positive blood culture

Jiang et al. 2020, China[14]	Randomized controlled trial	Not mentioned	D 100mg bd + R 600mg/d for 6 weeks vs. D 100mg bd + R 600mg/d for 6 weeks + L (IV) 400mg/d for 7 d	Failure: No clinical improvement or initial improvement followed by return of symptoms within 2 weeks<br>Relapse: Not defined	Clinical features + positive complement fixation test
Karabay et al., 2004, Turkey[15]	Open randomized study	Patients aged > 15 years	D 100mg bd + R 600mg/d for 45 d vs. O 400mg/d + R 600mg/d for 30 d	Failure: Not defined<br>Relapse: Reappearance of symptoms and signs and increasing titers of the serological tests and/or a positive culture during the follow-up	Clinical features + positive agglutination titre (≥1/160) and/or a positive culture
Karami et al., 2020, Iran[16]	Prospective randomized trial	Patients aged > 12 years	D 100mg bd + R 600mg/d for 8 weeks + G 5mg/kg/d for 7 days vs. 100mg bd + R 600mg/d for 12 weeks + G 5mg/kg/d for 7 days	Failure: Not defined<br>Relapse: Recurrence of clinical symptoms of brucellosis as confirmed by the serological tests [increased 2ME (IgG) and Wright titres compared to the post-treatment serology]	Clinical features + Wright titer>1:80; 2ME>1:40
Keramat et al., 2009, Iran[17]	Prospective randomized trial	Patients aged > 17 years	D 100mg bd plus R 15 mg/kg/daily (600–900 mg) for 8 weeks, vs. C 15 mg/kg/daily (500–750 mg twice a day) plus R 15 mg/kg/daily for 8weeks vs. C 15 mg/kg/daily (500–750 mg twice a day) plus D 100mg bd for 8 weeks. Those with spondylitis	Failure: symptoms and signs of the disease persisted or had increased at the end of eight to 12 weeks of therapy<br>Relapse: Reappearance of symptoms and signs of the disease accompanied by increasing titres [STAT	Clinical features + standard tube agglutination test (STAT) titer of antibodies to brucella >1/160 and 2-mercaptoethanol (2-ME) >1/80 and/or a positive blood culture

			had 4 extra weeks of treatment in all 3 arms	and 2-ME (IgG)] of the serological tests	
Liang et al., 2018, China[18]	Randomized controlled clinical trial	Not mentioned	D 100mg /d + R 900mg/d for 4 weeks vs. D 100mg /d + R 900mg/d + L 400mg/d for 4 weeks	Failure: No clinical improvement<br>Relapse: Not defined	Clinical features+ WHO diagnostic criteria [10]
Liu et al, 2018, China[19]	Randomized controlled clinical trial	Not mentioned	D 200mg/d for 12 weeks vs. D 200mg/d + R 900mg for 12 weeks	Failure: No clinical improvement<br>Relapse: Not defined	Clinical features + positive standard agglutination test
Liu et al, 2019, China[20]	Randomized controlled clinical trial	Patients aged > 16 years	D 100mg bd + R 600 to 900 mg/d for 6 weeks vs. D 100mg bd + R 600mg/d for 6 weeks + SM 500mg/d for 12 weeks	Failure: No clinical improvement<br>Relapse: Not defined	Clinical diagnosis + China CDC criteria[10]
Liu et al. 2021, China[21]	Randomized controlled clinical trial	Not mentioned	D 100mg bd + R 450mg /d for 6 weeks vs. D 100mg bd + L 500mg/d for 6 weeks	Failure: No clinical improvement	Clinical diagnosis + Complement fixation test
Majzoobi et al., 2022, Iran[22]	Single blind randomized trial	Patients aged > 18 years	D 100mg bd + hydroxychloroquine 400 mg daily for 4 weeks, and S 1 g daily for 3 weeks vs. D 100mg bd + hydroxychloroquine 400 mg daily for 6 weeks, and S 1 g daily for 3 weeks	Failure: not defined<br>Relapse: Recurrence of Clinical symptoms with increase in 2ME (IgG) titre	Clinical features + positive serology including standard tube agglutination (Wright) test ≥ 1/160 and 2-mercaptoetanol (2ME) ≥ 1/80
Mile et al., 2012, North Macedonia[23]	Prospective non-randomized trial	Patients aged > 8 years	D 100mg bd and R 900mg/d for 45 d + G 240mg/d for 7-10d vs. D 100mg bd and R 900mg/d for 45 d	Failure: Absence of or a weak tendency for improvement of symptoms and signs after 45 days	Clinical features + anti-Brucella antibody titres >1/320 or a demonstration of an at least fourfold rise in

				Relapse: Reappearance of symptoms and signs after treatment was completed	antibody titres in serum specimens obtained 3–4 weeks apart
Ranjbar et al., 2007, Iran[24]	Open randomized study	Patients aged > 8 years	D 100mg bd and R 10mg/kg/d for 8 weeks vs. D 100mg bd and R 10mg/kg/d for 8 weeks + amikacin 15mg/kg/d IM for 7 days	Failure: Symptoms or signs of the disease persisted at the end of treatment<br>Relapse: Reappearance of symptoms or signs of the disease with 2-ME test (IgG) or new positive blood cultures after therapy.	Clinical features + STA > 160 or culture positivity or 4-fold increase in wright titer in 2 weeks<br>All patients had a 2-ME wright test at 2 and 6 months of follow up.
Roushan et al., 2010, Iran[25]	Randomized unblinded trial	Patients aged > 10 years	D 100mg bd for 8 weeks + G 5mg/kg daily for 5d vs. IM S 1g for 2weeks + D 100mg bd for 45 d	Failure: Persistence or worsening of symptoms or signs at the end of treatment, as judged clinically<br>Relapse: Symptoms and signs of brucellosis reappeared and reduced titres of STA and 2-ME (IgG) increased again, or the Brucella species was isolated from blood culture during the follow-up period	Clinical features + STA >1:320 and 2ME titre >1:160 and / or positive culture
Roushan et al., 2004, Iran[12]	Randomized unblinded trial	Patients aged > 10 years	D 100mg bd and cotrimoxazole 8mg/kg/d for 8 weeks vs. R 15mg/kg/d and cotrimoxazole 8mg/kg/d for 8 weeks	Failure: Symptoms or signs of the disease persisting at the end of treatment<br>Relapse: Clinical features reappear and STAT, and 2 ME and	Clinical features + ≥1/320 standard tube agglutination titer (STAT) of antibodies to brucella with a 2-mercaptoethanol (2 ME) titre ≥1/160,

				brucella specific IgG titers increased	
Sha et al. 2017, China[26]	Randomized controlled trial	Not mentioned	D 100mg bd + R 600mg/d for 6 weeks vs. D 100mg bd + R 600mg/d + L 400mg/d for 6 weeks	Failure: No improvement in clinical symptoms or an initial improvement followed by recurrence of symptoms within 2 weeks	Clinical Diagnosis: China CDC criteria
Solera et al., 2004, Spain[27]	Prospective, double blind randomized trial	Patients aged > 18 years	D 100mg bd for 30 days + G 240mg IM/d for 7 days vs. D 100mg bd for 45 days + G 240mg IM/d for 7 days	Failure: Signs and symptoms persist by day 30 of treatment<br>Relapse: Positive blood culture or reappearance of signs and symptoms during the 12 months follow-up	Clinical features + STA titre > 1:160 or culture positivity, or 4-fold increase in antibody titre in a two-week interval
Taghvaee et al., 2011, Iran[28]	Prospective observational study	Patients aged > 15 years	D 100mg bd + R 600mg/d for 8 weeks vs. C 1g /d + R 600mg/d for 6 weeks	Failure: Not defined<br>Relapse: Not defined but 2-ME (IgG) titre done for all patients at 1-month post-treatment and at end of follow up	Clinical features + Wright and 2ME tests with titers equal to or more than 1/160 and 1/40 respectively
Saltoglu et al., 2002, Turkey[29]	Prospective observational study	Patients aged > 15 years	D 100mg bd + R 600mg/d for 45d vs. O 200mg bd + R 600mg/d for 6 weeks	Definition of failure: Not defined<br>Definition of relapse: Reappearance of symptoms and signs or increase in STA or both	Clinical features + 4-fold rise of brucella specific antibodies 2 weeks apart or significantly high titer (>1/160) in the standard tube agglutination test (STA) or positive culture

Salehi et al., 2023, Iran[30]	Open randomized study	Patients aged > 14 years	D 100mg bd + R 600mg/d for 8 weeks vs. D 100mg bd for 8 weeks + R 900 - 1200mg for 4 weeks and then R 600mg/d for 4 weeks	Definition of failure: Signs and symptoms of the disease present in the eighth week of treatment<br>Definition of relapse: Return of signs and symptoms after therapy supported by positive serology (2-ME titre tested for all patients at the end of follow up)	Clinical features + standard tube agglutination titre ≥1/160 or Coombs Wright titre ≥1/320 or 2-ME titre > 1/160 positive culture or PCR test
Sofian et al., 2014, Iran[31]	Randomized unblinded trial	Patients aged > 9 years	D 100mg bd + R 600mg/d for 6 weeks with IM S 1g for 7d vs. D 100mg bd + R 600mg/d for 8weeks with IM S 1g for 7d	Definition of failure: Reappearance of brucellosis signs and symptoms and a rise in antibody titers at the end of treatment<br>Definition of relapse: Reappearance of signs and symptoms and a rise in antibody titres during the follow-up.	Clinical features + standard tube agglutination test (STA) > 1:160, and 2-mercaptoethanol (2ME) agglutination > 1:80
Sun et al. 2020, China[32]	Randomized clinical trial	Not mentioned	D 100mg bd + R 600mg/d for 6 weeks vs. D 100mg bd + R 600mg/d + L 500mg/d for 6 weeks	Definition of failure: No clinical improvement and positive RBPT<br>Relapse: not defined	Clinical features + Rose Bengal Plate test (RBPT)
Yin et al., 2015, China[33]	Randomized controlled trial	Patients aged > 16 years	D 100mg bd + R 600mg/d for 6 weeks vs. D 100mg bd + R 600mg/d for 6 weeks + L (IV) 400mg/d for 7 d	Definition of failure: Continued clinical symptoms and fever. Relapse was not defined.	Clinical features + positive complement fixation test

Zhang et al. 2022, China[34]	Randomized controlled trial	Not mentioned	D 100mg bd + R 900mg/d for 6 weeks vs. D 100mg bd + R 900mg/d + L 500mg/d for 6 weeks	Definition of failure: Continued clinical symptoms with positive Brucella culture	Clinical features + Rose Bengal Plate test (RBPT) with or without culture

*Abbreviations for antibiotics: D – Doxycycline, R – Rifampicin, C – Ciprofloxacin, O – Ofloxacin, G – Gentamycin, S – Streptomycin, L -Levofloxacin, SM – Sulfamethoxazole, T – Tetracycline, Other abbreviations: bd (*bis in die*) – Twice daily, IM – intramuscular, IV - intravenous ME – Mercaptoethanol, MRI – Magnetic resonance imaging, STAT - standard tube agglutination titre,

Supplementary Table 3. Characteristics of excluded studies

**Study**	**Reason for exclusion**
Ozturk-Engin et al., 2014[35]	Not a controlled clinical study
Hashemi et al., 2011[36]	Conference abstract only – the full text published in the following year is included
Solera et al., 1997[37]	Conference abstract only
Ahmadvand et al., 2021[38]	Wrong intervention (Vitamin A)
Akova et sl., 1993[39]	Outside the time limit of 25 years
Al Anazi et al., 2012[40]	Wrong intervention (levamisole)
Barrier et al., 1994[41]	Outside the time limit of 25 years
Berdalieva et al., 2015[42]	Full text not found
Colmenero et al., 1994[43]	Outside the time limit of 25 years
Chen et al. 2016[44]	Outcomes were not clearly defined
Chen et al. 2016[45]	Laboratory diagnostic criteria were not clear
Deng et al. 2015[46]	Antibiotic doses are not clearly mentioned
Duisenova et al., 2002[47]	Full text not found
Duĭsenova et al., 2002[48]	Full text not found
Erdem et al., 2012[49]	Wrong study design
Geyik et al. 2003[50]	Animal study
Hashemi et al., 2011[36]	Conference abstract
Jafari et al., 2015[51]	Wrong intervention (Celecoxib)
Jia et al., 2023[52]	Wrong intervention
Ju et al., 2022[53]	Full-text not available
Kalo et al., 1996[54]	Outside the time limit of 25 years
Khuri-Bulos et al., 1993[55]	Outside the time limit of 25 years
Majzoobi et al., 2018[56]	Wrong intervention (Hydroxychloroquine)
Mert et al., 1996[57]	Outside the time limit of 25 years
Montejo et al., 1993[58]	Outside the time limit of 25 years
Pappas et al., 2004[59]	Wrong study design
Printzis et al., 1994[60]	Outside the time limit of 25 years
Roushan et al., 2009[61]	Conference abstract
Sarmadian et al., 2009[62]	Conference abstract
Shen et al., 2021[63]	Antibiotic doses not mentioned

Shen et al., 2018[64]	Number of initial cure and failures not clearly reported
Shul'diakov et al., 2011[65]	Wrong intervention (Cytoflavin)
Smagina et al., 2011[66]	Wrong intervention (Cycloferon)
Soleimani et al., 2021[67]	Wrong intervention (Zinc supplementation)
Solera et al., 1997[37]	Outside the time limit of 25 years
Solera et al., 1995[68]	Outside the time limit of 25 years
Solera et al., 1996[69]	Outside the time limit of 25 years
Solera et al., 1994[70]	Wrong study design – a meta-analysis and outside of time limit of 25 years
Sun et al. 2015[71]	Criteria for confirmation of Brucellosis not mentioned.
Tian et al. 2009[72]	The antibiotic dosing regimen was not clear
Yang et al., 2008[73]	Wrong comparator
Yangbin et al., 2017[74]	Wrong intervention (Surgical Management)
Zamani et al., 2022[75]	Wrong intervention (Probiotics Supplementation)
Zhang et al. 2019[76]	Numbers for cure / failures or relapses not clearly reported

Supplementary Table 4. Risk of bias of Included studies

Study	Comments on risk of bias*
Agalar et al., 1999[1]	The study was randomized but allocation concealment was not mentioned (selection bias – intermediate risk). The participants and assessors were not blinded (performance and detection bias – high risk). Outcomes were recorded for all participants in each trial arm (attrition bias – low risk). No other risks of bias identified.
Alavi et al., 2007[2]	The study was randomized but the details on allocation concealment and blinding were not mentioned (selection bias, performance bias, detection bias – all unclear risk). Attrition rate was reported to be less than 10% (attrition bias – low risk). However, infection or relapse was not confirmed by culture.
Alikhani et al., 2007[3]	The method of randomization was mentioned but allocation concealment was not mentioned (selection bias – intermediate risk). The study was single blind (performance bias – low risk) (detection bias – high risk) and a less than 10% attrition rate was reported (attrition bias – low risk). However, the participant characteristics at baseline were not mentioned or compared (other bias – high risk).
Alp et al., 2006[4]	The study was a “non-randomized” open label controlled clinical study (selection bias – high risk). There was no blinding process (performance bias, detection bias – high risk). Attrition rate was reported to be less than 10% (attrition bias – low risk). No other risk of bias identified.
Bayindir et al., 2003[5]	The study was a prospective randomized trial, but allocation concealment was not mentioned (selection bias – intermediate risk). The study was not blinded (performance bias – high risk) (detection bias – high risk). All participants were accounted for at the end of the study (attrition bias – low risk). No other risks of bias were identified.
Didgar et al., 2012[6]	The study was randomized, and the allocation concealment was reported (selection bias – low risk). The study was triple blinded (performance bias – low risk) (detection bias – low risk), but a more than 10% attrition rate was reported (attrition bias – high risk). There was no follow up at the end of the study to report on relapses.
El-Miedany et al., 2003[7]	The study was not randomized. (selection bias – high risk) or blinded (performance bias – high risk) (detection bias – high risk). Attrition rate was more than 10% for 24 months and relapse data was not available for 49 participants (attrition

	bias – high risk). Data was presented for only those who completed the 24 month follow up (reporting bias – high risk). The participants were not treated for a specific period and the mean duration of treatment was not provided for each treatment regimen (high risk of bias).
Ersoy et al., 2005[8]	Randomized trial, but the allocation concealment was not reported (selection bias – high risk). Unblinded study (performance bias – high risk) (detection bias – high risk). The attrition rate was reported to be less than 10% (attrition bias – low risk). Outcomes were not reported for 10 participants that did not attend the 6 month follow up (reporting bias – high risk). The definition of cure is this study is unclear, it is unclear if 2-ME titre was used to detect relapses (Other biases - high risk).
Feng et al., 2016[9]	The study was not randomized, and allocation concealment was not mentioned (selection bias – high risk), Blinding is not mentioned (performance bias and detection bias – unclear risk), Attrition bias is low risk as all participants are accounted for the outcome of cure. However, there is a high risk of reporting bias as though participants were followed up for relapses, the numbers are not reported. The study reports two categories of people as "cures" and "improved with treatment". Since the latter was not explicitly mentioned as failures we did not include this "intermediate" outcome as a treatment failure in the meta-analysis but this introduces a high risk of bias in interpreting and reporting of the results (Other bias – high risk).
Hasanain et al., 2016[11]	Randomized trial but the allocation concealment was not mentioned (selection bias – intermediate risk). No mention of blinding (performance bias – unclear risk) (detection bias – high unclear). Incomplete data for 13 participants; >10% attrition rate (attrition bias – high risk). Definition of cure is not clear, and the diagnosis was not confirmed by culture, it is unclear if 2-ME titre was used to detect relapses (Other biases - high risk).
Hashemi et al., 2012[13]	The study was a prospective randomized clinical trial, but the allocation concealment was not reported. (selection bias – intermediate risk). The study was not blinded (performance bias – high risk) (detection bias – high risk). The attrition rate was reported to be less than 10% (attrition bias – low risk). No other risks of bias identified.

Jiang et al., 2020[14]	Participant selection was randomized, but allocation concealment was not mentioned (selection bias – intermediate risk). Blinding is not mentioned (performance bias and detection bias – unclear risk), Attrition bias is low risk as all participants are accounted for the outcome of cure. However, there is a high risk of reporting bias as though participants were followed up for relapses, the numbers are not reported. The study reports two categories of people as "cures" and "improved with treatment". Since the latter was not explicitly mentioned as failures we did not include this "intermediate" outcome as a treatment failure in the meta-analysis but this introduces a high risk of bias in interpreting and reporting of the results (Other bias – high risk).
Karabay et al., 2004[15]	The participants were randomly assigned but the allocation concealment was not mentioned (selection bias – intermediate risk). Open label study (performance bias – high risk) (detection bias – high risk). The attrition rate was more than 10% (attrition bias – high risk). Cure and failure rates are not reported (reporting bias – high risk). Follow up period is not specified, and the sample size was not met due to attrition (Other biases - high risk).
Karami et al., 2020[16]	Participant selection was randomized, but allocation concealment was not mentioned (selection bias – intermediate risk). Blinding was not possible due to the differences in the duration of treatment (performance bias – high risk) (detection bias – high risk). The authors do not describe if all participants randomized completed follow up (attrition bias – unclear risk). Total number of treatment successes and failures are not reported (reporting bias – high risk). Diagnosis and treatment success was not confirmed by culture (high risk of bias)
Keramat et al., 2009[17]	Randomized study but allocation concealment was not done (selection bias – high risk). The study was not blinded as some participants were treated for an extra 8 weeks (performance bias – high risk) (detection bias – high risk). The attrition rate was more than 10% (attrition bias – high risk).
Liang et al., 2018[18]	Participant selection was randomized, but allocation concealment was not mentioned (selection bias – intermediate risk). Blinding is not mentioned (performance bias and detection bias – unclear risk), Attrition bias is low risk as all participants are accounted for the outcome of cure. Reporting bias is low as all outcomes assessed were reported. The study reports two categories of people as "cures" and "improved with treatment". Since the latter was not explicitly mentioned as

	failures, we did not include this “intermediate” outcome as a treatment failure in the meta-analysis but this introduces a high risk of bias in interpreting and reporting of the results (Other bias – high risk).
Liu et al., 2018[19]	Participant selection was randomized, but allocation concealment was not mentioned (selection bias – intermediate risk). Blinding is not mentioned (performance bias and detection bias – unclear risk), Attrition bias is low risk as all participants are accounted for the outcome of cure. Reporting bias is high risk as the relapses were meant to be assessed but not reported. The study reports two categories of people as “cures” and “improved with treatment”. Since the latter was not explicitly mentioned as failures, we did not include this “intermediate” outcome as a treatment failure in the meta-analysis, but this introduces a high risk of bias in interpreting and reporting of the results (Other bias – high risk).
Liu et al., 2019[20]	Participant selection was not randomized, and allocation concealment was not mentioned (selection bias – high risk). Blinding is not mentioned (performance bias and detection bias – unclear risk), Attrition bias is low risk as all participants are accounted for the outcome of cure. Reporting bias is high risk as the relapses were meant to be assessed but not reported. The study reports two categories of people as “cures” and “improved with treatment”. Since the latter was not explicitly mentioned as failures, we did not include this “intermediate” outcome as a treatment failure in the meta-analysis but this introduces a high risk of bias in interpreting and reporting of the results (Other bias – high risk).
Liu et al., 2021[21]	Participant selection was not randomized, and allocation concealment was not mentioned (selection bias – high risk). Blinding was not possible due to study design (performance bias and detection bias – high risk), Attrition bias is low risk as all participants are accounted for the outcome of cure. Reporting bias is high risk as relapses were not reported. No other risks of bias.
Majzoobi et al., 2022[22]	Randomized clinical trial but the allocation concealment was not mentioned (selection bias – intermediate risk). The study was a single blind study (performance bias – high risk) (detection bias – high risk). The attrition rate was more than 10% (attrition bias – high risk). Cure was based on clinical improvement only and therapeutic failure was not defined (Other biases - high risk).

Mile et al., 2012[23]	Non-randomized (selection bias – high risk), open label study (performance bias – high risk), with one arm receiving intravenous antibiotics (detection bias – unclear risk). The attrition rate was more than 10% (attrition bias – high risk). Relapse was only detected with re-appearance of symptoms (Other biases – high risk)
Ranjbar et al., 2007[24]	Randomized study but allocation concealment was not mentioned (selection bias – intermediate risk). Open label study with one arm having intravenous antibiotics (performance bias – high risk) (detection bias – high risk). The attrition rate was less than 10% (attrition bias – low risk). The cure rate and the therapeutic failure rate was not reported (reporting bias – high risk). Blood culture was not done for all suspected cases (Other biases - high risk).
Roushan et al., 2004[77]	Randomized trial with allocation concealment described (selection bias – low risk). Open label study (performance bias – high risk) (detection bias – high risk). The attrition rate was reported to be less than 10% (attrition bias – low risk). No other risks of bias were identified.
Roushan et al., 2010[25]	Randomized study with allocation concealment described (selection bias – low risk). Unblinded study (performance bias – high risk) (detection bias – high risk). The attrition rate was reported to be less than 10% (attrition bias – low risk). No other risks of bias were identified.
Roushan et al., 2006[12]	Randomized comparative study with allocation concealment described (selection bias – low risk). No mention of blinding – study described as a comparatives study (performance bias – unclear risk) (detection bias – unclear risk). The attrition rate was reported to be less than 10% (attrition bias – low risk). All patients were accounted for including those not completing follow up (reporting bias – low risk). No other risks of bias were identified.
Salehi et al., 2023[30]	Randomized study but allocation concealment was not mentioned (selection bias – intermediate risk). Open label study (performance bias – high risk) (detection bias – high risk). The attrition rate was reported to be more than 10% (attrition bias – high risk). No other risks of bias were identified.
Saltoglu et al., 2002[29]	Not a randomized or blinded study (selection bias – high risk, performance and detection biases – high risk). It is not clear if all enrolled patients were followed up for 6 months (attrition bias – unclear risk). Treatment success and failure

	was not reported (reporting bias – high risk). No sample size calculation provided and it is unclear if 2-ME titre was used to detect relapses (Other biases - high risk).
Sha et al., 2017[26]	Participant selection was randomized, but allocation concealment was not mentioned (selection bias – intermediate risk). Blinding is not mentioned (performance bias and detection bias – unclear risk), Attrition bias is low risk as all participants are accounted for the outcome of cure. Reporting bias is low as all outcomes assessed were reported. The study reports two categories of people as “cures” and “improved with treatment”. Since the latter was not explicitly mentioned as failures, we did not include this “intermediate” outcome as a treatment failure in the meta-analysis but this introduces a high risk of bias in interpreting and reporting of the results (Other bias – high risk).
Sofian et al., 2014[31]	Randomized study but allocation concealment was not mentioned (selection bias – intermediate risk). Unblinded trial (performance bias – high risk) (detection bias – high risk). The attrition rate was reported to be less than 10% (attrition bias – low risk). No other risks of bias were identified.
Solera et al., 2004[27]	Randomized study but allocation concealment was not mentioned (selection bias – intermediate risk). The study was double blinded (performance bias – low risk) (detection bias – low risk). The attrition rate was more than 10% (attrition bias – high risk). Total cure and therapeutic failures were not mentioned (reporting bias – high risk). No other risks of bias were identified.
Sun et al., 2020[32]	Participant selection was randomized, but allocation concealment was not mentioned (selection bias – intermediate risk). Blinding is not mentioned (performance bias and detection bias – unclear risk), Attrition bias is low risk as all participants are accounted for the outcome of cure. Reporting bias is low as all outcomes assessed were reported. The study reports two categories of people as “cures” and “improved with treatment”. Since the latter was not explicitly mentioned as failures, we did not include this “intermediate” outcome as a treatment failure in the meta-analysis but this introduces a high risk of bias in interpreting and reporting of the results (Other bias – high risk).
Taghvee et al., 2011[28]	Not a randomized or blinded study (selection bias – high risk) (performance bias – high risk) (detection bias – high risk). The attrition rate was reported to be less than 10% (attrition bias – low risk). It is not clear if all enrolled participants were

	followed up until the completion of the study (reporting bias – unclear risk). Sample size calculation not mentioned, and infection was not confirmed with culture (Other biases - high risk).
Yin et al., 2015[33]	Participant selection was randomized, but allocation concealment was not mentioned (selection bias – intermediate risk). Double blinded study (performance bias and detection bias – low risk), All participants are accounted for the outcome of cure (Attrition bias – low risk) . Reporting bias is high as relapses were not reported. No other risks of bias noted.
Zhang et al. 2022[34]	Participant selection was randomized, but allocation concealment was not mentioned (selection bias – intermediate risk). Blinding is not mentioned (performance bias and detection bias – unclear risk), Low risk of attrition and reporting bias. The study reports two categories of people as “cures” and “improved with treatment”. Since the latter was not explicitly mentioned as failures, we did not include this “intermediate” outcome as a treatment failure in the meta-analysis but this introduces a high risk of bias in interpreting and reporting of the results (Other bias – high risk).

Supplementary Table 5. Severe adverse events (SAE) reported in each study (only studies that reported SAE are shown)

Study	Treatment group	Number of patients with SAE	Notes
Alavi et al. 2007[2]	Doxycycline + Rifampicin	0	
	Doxycycline + Cotrimoxazole	2	Treatment stopped due to SAE
Alp et al., 2006[4]	Doxycycline + Streptomycin	0	
	Ciprofloxacin + Rifampicin	2	
Ersoy et al., 2005[8]	Ofloxacin + Rifampicin	1	
	Doxycycline + Rifampicin	2	
	Doxycycline + Streptomycin	0	
Mile et al., 2012[23]	Doxycycline + Rifampicin + Gentamycin	4	
	Doxycycline + Rifampicin	3	
Roushan et al., 2004[78]	Doxycycline + Cotrimoxazole	2	
	Rifampicin + Cotrimoxazole	5	
Sofian et al., 2014[31]	Doxycycline + Rifampicin + Streptomycin	3	
	Doxycycline + Rifampicin + Streptomycin	2	

References

1. Agalar C, Usubutun S, Turkyilmaz R. Ciprofloxacin and rifampicin versus doxycycline and rifampicin in the treatment of brucellosis. Eur J Clin Microbiol Infect Dis. 1999;18(8):535-8. doi: 10.1007/s100960050344. PubMed Central PMCID: PMC10517189.
2. Alavi SM, Rajabzadeh AR. Comparison of two chemotherapy regimen: Doxycycline-rifampicin and doxycycline cotrimoxazol in the brucellosis patients Ahvaz, Iran, 2004-2006. Pak J Med Sci. 2007;23(6):889-92.
3. Alikhani A, Heidarzadeh A. A new therapeutic management in acute uncomplicated brucellosis: Comparison between three month-ofloxacin and doxycycline-based regimens. J Isfahan Med Sch. 2007;25(85).
4. Alp E, Koc RK, Durak AC, Yildiz O, Aygen B, Sumerkan B, et al. Doxycycline plus streptomycin versus ciprofloxacin plus rifampicin in spinal brucellosis [ISRCTN31053647]. BMC Infect Dis. 2006;6. doi: 10.1186/1471-2334-6-72. PubMed Central PMCID: PMC16606473.

5. Bayindir Y, Sonmez E, Aladag A, Buyukberber N. Comparison of five antimicrobial regimens for the treatment of brucellar spondylitis: a prospective, randomized study. J Chemother. 2003;15(5):466-71. Epub 2003/11/06. doi: 10.1179/joc.2003.15.5.466. PubMed PMID: 14598939.
6. Didgar F, Sarmadian H, Zarin Far N, Rafiee M, Choghae M. Comparison between efficacy of ciprofioxacin-doxycycline and rifampin-doxycycline regimens in treatment of brucellosis. J Zanjan Univ Med Sci Health Serv. 2012;20(80):2.
7. El Miedany YM, El Gaafary M, Baddour M, Ahmed I. Human brucellosis: do we need to revise our therapeutic policy? J Rheumatol. 2003;30(12):2666-72. Epub 2004/01/14. PubMed PMID: 14719211.
8. Ersoy Y, Sonmez E, Tevfik MR, But AD. Comparison of three different combination therapies in the treatment of human brucellosis. Trop Doct. 2005;35(4):210-2. Epub 2005/12/16. doi: 10.1258/004947505774938765. PubMed PMID: 16354469.
9. Feng Z, Ma J. Study on the Clinical Application Value of Doxycycline Combined with Rifampicin in the Treatment of Chronic Brucella Arthritis and Its Effect on Pain [In Chinese]. Clinical Medical Research and Practice. 2016;1(21):65-6.
10. Jiang H, Feng L, Lu J. Updated Guidelines for the Diagnosis of Human Brucellosis - China, 2019. China CDC Wkly. 2020;2(26):487-9. Epub 2020/06/26. doi: 10.46234/ccdcw2020.129. PubMed PMID: 34594685; PubMed Central PMCID: PMCPMC8393123 interest.
11. Hasanain A, Mahdy R, Mohamed A, Ali M. A randomized, comparative study of dual therapy (doxycycline-rifampin) versus triple therapy (doxycycline-rifampin-levofloxacin) for treating acute/subacute brucellosis. Braz J Infect Dis. 2016;20(3):250-4. Epub 2016/04/19. doi: 10.1016/j.bjid.2016.02.004. PubMed PMID: 27086734; PubMed Central PMCID: PMCPMC9425512.
12. Roushan MRH, Mohraz M, Hajiahmadi M, Ramzani A, Valayati AA. Efficacy of gentamicin plus doxycycline versus streptomycin plus doxycycline in the treatment of brucellosis in humans. CLIN INFECT DIS. 2006;42(8):1075-80. doi: 10.1086/501359. PubMed Central PMCID: PMC16575723.
13. Hashemi SH, Gachkar L, Keramat F, Mamani M, Hajilooi M, Janbakhsh A, et al. Comparison of doxycycline-streptomycin, doxycycline-rifampin, and ofloxacin-rifampin in the treatment of brucellosis: a randomized clinical trial. Int J Infect Dis. 2012;16(4):e247-51. Epub 2012/02/03. doi: 10.1016/j.ijid.2011.12.003. PubMed PMID: 22296864.
14. Jiang L. Comparison of the efficacy of different antibacterial drug combination regimens in the treatment of brucellosis [In Chinese]. Chinese Journal of Clinical Rational Drug Use. 2020;13(10):53–4.
15. Karabay O, Sencan I, Kayas D, Sahin I. Ofloxacin plus rifampicin versus doxycycline plus rifampicin in the treatment of brucellosis: a randomized clinical trial [ISRCTN11871179]. BMC Infect Dis. 2004;4:18. Epub 2004/06/25. doi: 10.1186/1471-2334-4-18. PubMed PMID: 15214959; PubMed Central PMCID: PMCPMC459220.
16. Karami A, Mobaien A, Jozpanahi M, Moghtader-Mojdehi A, Javaheri M. Effect of 8-week and 12-week triple therapy (doxycycline, rifampicin, and gentamicin) on brucellosis: A comparative study. JOURNAL OF ACUTE DISEASE. 2020;9(4):161-5. doi: 10.4103/2221-6189.288594. PubMed PMID: WOS:000552143900005.
17. Keramat F, Ranjbar M, Mamani M, Hashemi SH, Zeraati F. A comparative trial of three therapeutic regimens: ciprofloxacin-rifampin, ciprofloxacin-doxycycline and doxycycline-rifampin in the treatment of brucellosis. Trop Doct. 2009;39(4):207-10. Epub 2009/09/19. doi: 10.1258/td.2009.090030. PubMed PMID: 19762571.
18. Liang C, Li J. The Influence of Different Antibiotic Combination Regimens on the Treatment Effect, Recurrence Rate, and Incidence of Adverse Reactions in Brucellosis [In Chinese]. Strait Pharmaceutical Journal. 2018;30(11):221-2.

19. Liu D. Clinical Efficacy Observation of Rifampicin Combined with Doxycycline in the Treatment of Acute Brucellosis [In Chinese]. Practical Medicine of China. 2018;13(04):77-9.
20. Liu J, Liu AJ, Mu WD. Efficacy of Doxycycline Combined with Compound Sulfamethoxazole in the Treatment of Acute Phase Brucellosis [In Chinese]. Laboratory Medicine and Clinical. 2019;16(22):3376-8.
21. Liu F, Du N. Efficacy observation of levofloxacin combined with doxycycline in the treatment of brucellosis [In Chinese]. Heilongjiang Medical Science. 2021;44(03):86-7.
22. Majzoobi MM, Hashmi SH, Emami K, Soltanian AR. Combination of doxycycline, streptomycin and hydroxychloroquine for short-course treatment of brucellosis: a single-blind randomized clinical trial. Infection. 2022;50(5):1267-71. Epub 2022/03/31. doi: 10.1007/s15010-022-01806-x. PubMed PMID: 35353333; PubMed Central PMCID: PMCPMC8966606.
23. Mile B, Valerija K, Krsto G, Ivan V, Ilir D, Nikola L. Doxycycline-rifampin versus doxycycline-rifampin-gentamicin in treatment of human brucellosis. Trop Doct. 2012;42(1):13-7. Epub 2012/02/01. doi: 10.1258/td.2011.110284. PubMed PMID: 22290107.
24. Ranjbar M, Keramat F, Mamani M, Kia AR, Khalilian FO, Hashemi SH, et al. Comparison between doxycycline-rifampin-amikacin and doxycycline-rifampin regimens in the treatment of brucellosis. Int J Infect Dis. 2007;11(2):152-6. Epub 2006/06/27. doi: 10.1016/j.ijid.2005.11.007. PubMed PMID: 16798042.
25. Roushan MRH, Amiri MJS, Janmohammadi N, Sadeghi Hadad M, Javanian M, Baiani M, et al. Comparison of the efficacy of gentamicin for 5 days plus doxycycline for 8 weeks versus streptomycin for 2 weeks plus doxycycline for 45 days in the treatment of human brucellosis: A randomized clinical trial. J Antimicrob Chemother. 2010;65(5):1028-35. doi: 10.1093/jac/dkq064. PubMed Central PMCID: PMC20215128.
26. Sha R. Comparison of different combination therapy regimens of antimicrobial drugs for brucellosis [In Chinese]. World Latest Medicine Information. 2017;17(58):127-8.
27. Solera J, Geijo P, Largo J, Rodriguez-Zapata M, Gijón J, Martinez-Alfaro E, et al. A randomized, double-blind study to assess the optimal duration of doxycycline treatment for human brucellosis. Clin Infect Dis. 2004;39(12):1776-82. Epub 2004/12/04. doi: 10.1086/426024. PubMed PMID: 15578399.
28. Taghvaee MRE, Nozadi MS, Hassani M. A comparison between doxycycline-rifampin and ciprofloxacin-rifampin regimens in the treatment of acute Brucellosis. Indian J Med Sci. 2011;65(10):436-43. doi: 10.4103/0019-5359.109263. PubMed Central PMCID: PMC23511044.
29. Saltoglu N, Tasova Y, Inal AS, Seki T, Aksu HS. Efficacy of rifampicin plus doxycycline versus rifampicin plus quinolone in the treatment of brucellosis. Saudi Med J. 2002;23(8):921-4. PubMed Central PMCID: PMC12235463.
30. Salehi M, Farbod F, Khalili H, Rahmani H, Jafari S, Abbasi A. Comparing efficacy and safety of high-dose and standard-dose rifampicin in the treatment of brucellosis: a randomized clinical trial. J Antimicrob Chemother. 2023;78(4):1084-91. Epub 2023/03/08. doi: 10.1093/jac/dkad051. PubMed PMID: 36880215.
31. Sofian M, Velayati A-A, Aghakhani A, McFarland W, Farazi A-A, Banifazl M, et al. Comparison of two durations of triple-drug therapy in patients with uncomplicated brucellosis: A randomized controlled trial. Scand J Infect Dis. 2014;46(8):573-7. doi: 10.3109/00365548.2014.918275. PubMed PMID: 97029021. Language: English. Entry Date: In Process. Revision Date: 20191111. Publication Type: Article. Journal Subset: Biomedical.
32. Sun L. Effects of triple therapy of Ofloxacin, Rifampicin and Doxycycline in treatment of patients with brucellosis [In Chinese]. Chinese People’s Health. 2020;32(24):20-1.

33. Yin M, Wang YH, Song Y, Li H, Zhang L, Li MH. Comparison of Different Antibiotic Combination Therapies for Brucellosis [In Chinese]. Chinese Journal of Experimental and Clinical Infectious Diseases (Electronic Edition). 2015;9(06):81-3.
34. Zhang C, Wang Z, Yang YJ, Li Y. Efficacy of rifampicin combined with doxycycline and levofloxacin in the treatment of brucellosis [In Chinese]. Medical Information. 2022;35(03):112-4.
35. Ozturk-Engin D, Erdem H, Gencer S, Kaya S, Baran AI, Batirel A, et al. Liver involvement in patients with brucellosis: Results of the Marmara study. Eur J Clin Microbiol Infect Dis. 2014;33(7):1253-62. doi: 10.1007/s10096-014-2064-4. PubMed Central PMCID: PMC24557334.
36. Hashemi S, Gachkar L, Keramat F, Mamani M, Hajilooi M, Janbakhsh A, et al. Comparison of doxycycline-streptomycin, Doxycyclinerifampin and ofloxacin-rifampin in the treatment of human brucellosis. Clin Microbiol Infect. 2011;17(SUPPL. 4):S441-S2. doi: https://dx.doi.org/10.1111/j.1469-0691.2011.03558.x.
37. Solera J, Espinosa A, Martínez-Alfaro E, Sánchez L, Geijo P, Navarro E, et al. Treatment of human brucellosis with doxycycline and gentamicin. Antimicrob Agents Chemother. 1997;41(1):80-4. Epub 1997/01/01. doi: 10.1128/aac.41.1.80. PubMed PMID: 8980759; PubMed Central PMCID: PMCPMC163664.
38. Ahmadvand N, Zarinfar N, Soofian M. The Effectiveness of Vitamin A on the Symptoms of Brucellosis. J Babol Univ Med Sci. 2021;23(1):252-8. doi: 10.22088/jbums.23.1.252.
39. Akova M, Uzun O, Akalin HE, Hayran M, Unal S, Gür D. Quinolones in treatment of human brucellosis: comparative trial of ofloxacin-rifampin versus doxycycline-rifampin. Antimicrob Agents Chemother. 1993;37(9):1831-4. Epub 1993/09/01. doi: 10.1128/aac.37.9.1831. PubMed PMID: 8239591; PubMed Central PMCID: PMCPMC188077.
40. Al Anazi AR, Al Aska AK, Al Tuwaijri AS, Al-Orainey IO, Al-Hedaithy MA, Al Majid FM, et al. The effect of levamisole combined with standard treatment vs. standard treatment on the functions of polymorphonulcear cells and monocytes in patients with brucellosis. Clin Microbiol Infect. 2012;18(SUPPL. 3):62. doi: https://dx.doi.org/10.1111/j.1469-0691.2012.03801.x.
41. Barrier JH, Traore AK, Magadur-Joly G, Gassin M. Treatment of afocal chronic brucellosis with doxycycline-rifampicine during three months (21 cases). MED MAL INFECT. 1994;24(11):1181-3. doi: 10.1016/S0399-077X(05)81349-6.
42. Berdalieva FA. An effect of the components of etiotropic therapy of acute brucellosis on the development of relapses in patients. Infektsionnye Bolezni. 2015;13(3):18-22.
43. Colmenero JD, Fernández-Gallardo LC, Agúndez JA, Sedeño J, Benítez J, Valverde E. Possible implications of doxycycline-rifampin interaction for treatment of brucellosis. Antimicrob Agents Chemother. 1994;38(12):2798-802. Epub 1994/12/01. doi: 10.1128/aac.38.12.2798. PubMed PMID: 7695265; PubMed Central PMCID: PMCPMC188288.
44. Chen RL. Clinical Observation of Doxycycline Combined with Levofloxacin in the Treatment of Brucellosis [In Chinese]. Chinese Medical Guide. 2016;14(26):68.
45. Chen F, Ma J, Luo Y, Liao SL. Clinical Efficacy of Doxycycline-containing Drugs in the Treatment of Brucella Spondylitis [In Chinese]. Journal of Endemic Diseases Control. 2016;31(09):998.
46. Deng LL, Zhang JY. Clinical Efficacy Observation of Rifampicin Combined with Doxycycline in the Treatment of Brucellosis [In Chinese]. Chinese Journal of Women's Health Research. 2016;(09):222+1.

47. Duĭsenova AK, Kurmanova KB, Kurmanova GM. [Ciprofloxacin in the treatment of patients with brucellosis]. Antibiot Khimioter. 2002;47(10):3-7. Epub 2003/04/05. PubMed PMID: 12674788.
48. Duisenova AK, Kurmanova KB, Kurmanova GM. Experience of brucellosis treatment with ciprofloxacin. Antibiot Khimioter. 2002;47(10):3-7. PubMed Central PMCID: PMC12674788.
49. Erdem H, Ulu-Kilic A, Kilic S, Karahocagil M, Shehata G, Eren-Tulek N, et al. Efficacy and tolerability of antibiotic combinations in neurobrucellosis: Results of the Istanbul study. ANTIMICROB AGENTS CHEMOTHER. 2012;56(3):1523-8. doi: 10.1128/AAC.05974-11. PubMed Central PMCID: PMC22155822.
50. Geyik MF, Dikici B, Kokoglu OF, Bosnak M, Celen MK, Hosoglu S, et al. Therapeutic effect of spiramycin in brucellosis. Pediatr Int. 2003;45(1):31-4. Epub 2003/03/26. doi: 10.1046/j.1442-200x.2003.01672.x. PubMed PMID: 12654065.
51. Jafari S, Ashrafizadeh SG, Zeinoddini A, Rasoulinejad M, Entezari P, Seddighi S, et al. Celecoxib for the treatment of mild-to-moderate depression due to acute brucellosis: a double-blind, placebo-controlled, randomized trial. J Clin Pharm Ther. 2015;40(4):441-6. Epub 2015/05/27. doi: 10.1111/jcpt.12287. PubMed PMID: 26009929.
52. Jia YL, Zuo XH, Zhang Y, Yao Y, Yin YL, Yang XM. Clinical value and effectiveness profiles of oblique lateral interbody fusion and posterior lumbar interbody fusion in the treatment of lumbar brucellosis spondylitis. Eur Rev Med Pharmacol Sci. 2023;27(9):3854-63. Epub 2023/05/19. doi: 10.26355/eurrev_202305_32291. PubMed PMID: 37203810.
53. Ju LL, Zhu B, Li W. Clinical efficacy of doxycycline hydrochloride combined with rifampicin in the treatment of brucellosis [In Chinese]. Chinese Journal of Endemic Disease Control. 2022;37(05):439+41.
54. Kalo T, Novi S, Nushi A, Dedja S. Ciprofloxacin plus doxycycline versus rifampicin plus doxycycline in the treatment of acute brucellosis. MED MAL INFECT. 1996;26(SPEC. ISS. JUN.):587-9. doi: 10.1016/S0399-077X(96)80077-1.
55. Khuri-Bulos NA, Daoud AH, Azab SM. Treatment of childhood brucellosis: results of a prospective trial on 113 children. Pediatr Infect Dis J. 1993;12(5):377-81. Epub 1993/05/01. doi: 10.1097/00006454-199305000-00005. PubMed PMID: 8327297.
56. Majzoobi MM, Hashemi SH, Mamani M, Keramat F, Poorolajal J, Basir HRG. Effect of hydroxychloroquine on treatment and recurrence of acute brucellosis: a single-blind, randomized clinical trial. Int J Antimicrob Agents. 2018;51(3):365-9. doi: 10.1016/j.ijantimicag.2017.08.009. PubMed PMID: WOS:000427582000011.
57. Mert A, Dumankar A, Tabak F, Kurtoglu E, Ozturk R, Aktuglu Y. Comparative trial of tetracycline-streptomycin versus doxycycline-rifampin in treatment of our brucellosis cases. 10TH MEDITERRANEAN CONGRESS OF CHEMOTHERAPY1996. p. 261-4.
58. Montejo JM, Alberola I, Glez-Zarate P, Alvarez A, Alonso J, Canovas A, et al. Open, randomized therapeutic trial of six antimicrobial regimens in the treatment of human brucellosis. Clin Infect Dis. 1993;16(5):671-6. Epub 1993/05/01. doi: 10.1093/clind/16.5.671. PubMed PMID: 8507759.
59. Pappas G, Seitaridis S, Akritidis N, Tsianos E. Treatment of brucella spondylitis: Lessons from an impossible meta-analysis and initial report of efficacy of a fluoroquinolone-containing regimen. Int J Antimicrob Agents. 2004;24(5):502-7. doi: 10.1016/j.ijantimicag.2004.05.003. PubMed Central PMCID: PMC15519485.
60. Printzis S, Raptopoulou-Gigi M, Orphanou-Koumerkeridou H, Lagre F, Goulis G. Immunotherapy in chronic brucellosis. Effect of levamisole and interferon; mechanisms of action and clinical value. Immunopharmacol Immunotoxicol. 1994;16(4):679-93. Epub 1994/11/01. doi: 10.3109/08923979409019745. PubMed PMID: 7876467.

61. Roushan MRH, Amiri MJS, Janmohammadi N, Javanian M, Baiani M. Optimal duration of gentamicin containing regimen for the treatment of uncomplicated brucellosis. Clin Microbiol Infect. 2009;15(S4):S47-S8. doi: https://dx.doi.org/10.1111/j.1469-0691.2009.02857.x.
62. Sarmadian H, Didgar F, Sufian M, Zarinfar N, Salehi F. Comparison between efficacy of cipofoxacin doxycycline and rifampin - Doxycycline regimens in treatment and relapse of brucellosis. Trop Med Int Health. 2009;14(SUPPL. 2):209. doi: https://dx.doi.org/10.1111/j.1365-3156.2009.02354-2.x.
63. Shen L. Analysis of the efficacy of drug therapy alone for patients with atypical Brucella osteoarthritis. China Prac Med. 2021;16(31):128–30.
64. Shen Y. Clinical Efficacy of Doxycycline Combined with Rifampicin in the Treatment of Brucellosis [In Chinese]. Modern Medicine of China. 2018;12(14):163-4.
65. Shul'diakov AA, Liapina EP, Soboleva LA, Reshetnikov AA, Zubareva EV, Trubetskov AD, et al. [The use of cytoflavin for the treatment of chronic brucellosis]. Klinicheskaia meditsina. 2011;89(2):56-8. PubMed Central PMCID: PMC21574446.
66. Smagina AN, Shul'dyakov AA. Effect of immunomodulator cycloferon on life quality and psychoemotional state of patients with chronic active brucellosis on background of complex pharmacotherapy. Eksp Klinicheskaya Farmakol. 2011;74(2):39-43. PubMed Central PMCID: PMC21476285.
67. Soleimani Z, Niaparast H, Sharif MR, Moroji A. The effect of zinc supplementation on clinical symptoms of brucellosis patients (quasi-experimental study). Turkish Journal of Physiotherapy and Rehabilitation. 2021;32(3):4116-22.
68. Solera J, Rodríguez-Zapata M, Geijo P, Largo J, Paulino J, Sáez L, et al. Doxycycline-rifampin versus doxycycline-streptomycin in treatment of human brucellosis due to Brucella melitensis. The GECMEI Group. Grupo de Estudio de Castilla-la Mancha de Enfermedades Infecciosas. Antimicrob Agents Chemother. 1995;39(9):2061-7. Epub 1995/09/01. doi: 10.1128/aac.39.9.2061. PubMed PMID: 8540716; PubMed Central PMCID: PMCPMC162881.
69. Solera J, Espinosa A, Geijo P, Martínez-Alfaro E, Sáez L, Sepúlveda MA, et al. Treatment of human brucellosis with netilmicin and doxycycline. Clin Infect Dis. 1996;22(3):441-5. Epub 1996/03/01. doi: 10.1093/clinids/22.3.441. PubMed PMID: 8852960.
70. Solera J, Martinez-Alfaro E, Saez L. Meta-analysis of the efficacy of the combination of rifampicin and doxycyclin in the treatment of human brucellosis. MED CLIN. 1994;102(19):731-8. PubMed Central PMCID: PMC8041202.
71. Sun HT. Clinical Efficacy Observation of Rifampicin Combined with Doxycycline and Levofloxacin in the Treatment of Brucellosis [In Chinese]. Chinese Health Standard Management. 2015;6(22):123-4.
72. Tian JL, Qian LJ, Zhang MW, Zhang YH. Study on the Efficacy of High-Dose Levofloxacin-based Regimen in the Treatment of Acute Brucellosis [In Chinese]. Chinese Ethnic and Folk Medicine. 2009;18(16):121.
73. Yang XM, Shi W, Du YK, Yin YL, Meng XY, Zou YW. Investigation on the curative effect of brucellar spondylitis. Chin J Endemiol. 2008;27(6):699-703.
74. Yangbin C, Jun-Song Y, Tao L, Peng L, Tuan-Jiang L, Li-Min H, et al. One-stage Surgical Management for Lumbar Brucella Spondylitis by Posterior Debridement, Autogenous Bone Graft and Instrumentation: A Case Series of 24 Patients. Spine (03622436). 2017;42(19):E1112-E8. doi: 10.1097/BRS.0000000000002093. PubMed PMID: 125316109. Language: English. Entry Date: 20180829. Revision Date: 20210116. Publication Type: journal article.
75. Zamani N, Ganjy Z, Sharif MR, Taghavi Ardakani A, Kheirkhah D, Sayyah M, et al. The Effects of Probiotics Supplementation on Clinical Status and Biomarkers of Oxidative Damage and Inflammation in Children with Brucellosis: A Randomized, Double-Blind, and Placebo-Controlled Trial. Evid Based Complement Alternat Med. 2022;2022:2541117. Epub 2022/09/02. doi: 10.1155/2022/2541117. PubMed PMID: 36045651; PubMed Central PMCID: PMCPMC9423965.

76. Zhang SJ. The Effect of Rifampicin Combined with Doxycycline Hydrochloride in the Treatment of Acute Brucellosis [In Chinese]. Chinese Journal of Civilian Medicine. 2019;31(22):14-6.
77. Roushan MR, Gangi SM, Ahmadi SA. Comparison of the efficacy of two months of treatment with co-trimoxazole plus doxycycline vs. co-trimoxazole plus rifampin in brucellosis. Swiss Med Wkly. 2004;134(37-38):564-8. Epub 2004/11/20. doi: 10.4414/smw.2004.10665. PubMed PMID: 15551160.
78. Roushan MRH, Gangi SME, Ahmadi S. Comparison of the efficacy of two months of treatment with co-trimoxazole plus doxycycline vs co-trimoxazole plus rifampin in brucellosis. Swiss Med Wkly. 2004;134(37-38):564-8. PubMed PMID: WOS:000224946700005.
